# Puckering effects of 4-hy­droxy-l-proline isomers on the conformation of ornithine-free Gramicidin S

**DOI:** 10.1107/S2056989024007771

**Published:** 2024-08-09

**Authors:** Akiko Asano, Kanako Nakayama, Sakura Okada, Takuma Kato, Mitsunobu Doi

**Affiliations:** a Osaka Medical and Pharmaceutical University, 4-20-1 Nasahara, Takatsuki, Osaka 590-1094, Japan; Tokyo University of Science, Japan

**Keywords:** crystal structure, cyclic deca­peptide, gramicidin s, pyrrolidine ring, *cis/trans*-isomers, β-sheet

## Abstract

The cyclic peptide *cyclo*(Val-Leu-Leu-d-Phe-Pro)_2_ (peptide **1**) was specifically designed for structural chemistry investigations, drawing inspiration from Gramicidin S (GS). Previous studies have shown that Pro residues within **1** adopt a down-puckering conformation of the pyrrolidine ring. By incorporating fluoride-Pro with 4-*trans*/*cis*-isomers into **1**, an up-puckering conformation was successfully induced. In the current investigation, introducing hy­droxy­prolines with 4-*trans*/*cis*-isomer configurations (tHyp/cHyp) into **1** gave *cyclo*(Val-Leu-Leu-d-Phe-tHyp)_2_ methanol disolvate monohydrate, C_62_H_94_N_10_O_12_·2CH_4_O·H_2_O (**4**), and *cyclo*(Val-Leu-Leu-d-Phe-cHyp)_2_ monohydrate, C_62_H_94_N_10_O_12_·H_2_O (**5**), respectively. However, the puckering of **4** and **5** remained in the down conformation, regardless of the geometric position of the hydroxyl group. Although the backbone structure of **4** with *trans*-substitution was asymmetric, the asymmetric backbone of **5** with *cis*-substitution was unexpected. It is speculated that the anti­cipated influence of stress from the geometric positioning, which was expected to affect the puckering, may have been mitigated by inter­actions between the hydroxyl groups of hy­droxy­proline, the solvent mol­ecules, and peptides.

## Abstract 2

The structures of Orn-free Gramicidin S with *cis/trans*-isomers of Hyp were solved, and the puckering of Hyp was unexpectedly down in both isomers.

## 1. Chemical context

Gramicidin S (GS) is a cyclic deca­peptide (Gause & Brazhnikova, 1944 ▸), forming intra­molecular sheet and turn structures (Hodgkin & Oughton, 1957 ▸; Schmidt *et al.*, 1957 ▸). Its chemical structure exhibits *C*2 symmetry, featuring a repeated sequence of Val-Orn-Leu-d-Phe-Pro (Balasubramanian, 1967 ▸). Our focus has been on investigating *cyclo*(Val-Leu-Leu-d-Phe-Pro)_2_ (peptide **1**), which mimics the structural characteristics of GS and facilitates structural analysis (Asano *et al.*, 2019 ▸, 2021 ▸). This is achieved by substituting the Orn residue with Leu to reduce inter­actions with solvent mol­ecules. In previous crystal structures of GS derivatives, the pyrrolidine ring of Pro consistently exhibited a down-puckering conformation (Doi *et al.*, 2001 ▸; Llamas-Saiz *et al.* 2007 ▸; Asano & Doi, 2019 ▸). To explore this further, we introduced a geometric isomer of 4-fluoride Pro into peptide **1**, examining its impact on Pro puckering and structure (Asano *et al.*, 2023 ▸). Notably, derivative **2** containing 4-*trans*-fluoro-Pro (tFPro) displayed an up-puckering for the first time, while derivative **3** with 4-*cis*-fluoro-Pro (cFPro) retained the conventional down-puckering. In this study, we introduced 4-*trans*-hy­droxy­proline (tHyp) and 4-*cis*-hy­droxy­proline (cHyp) into peptide **1**, resulting in derivatives **4** and **5**, respectively. We compared their structures with fluoride-Pro derivatives.
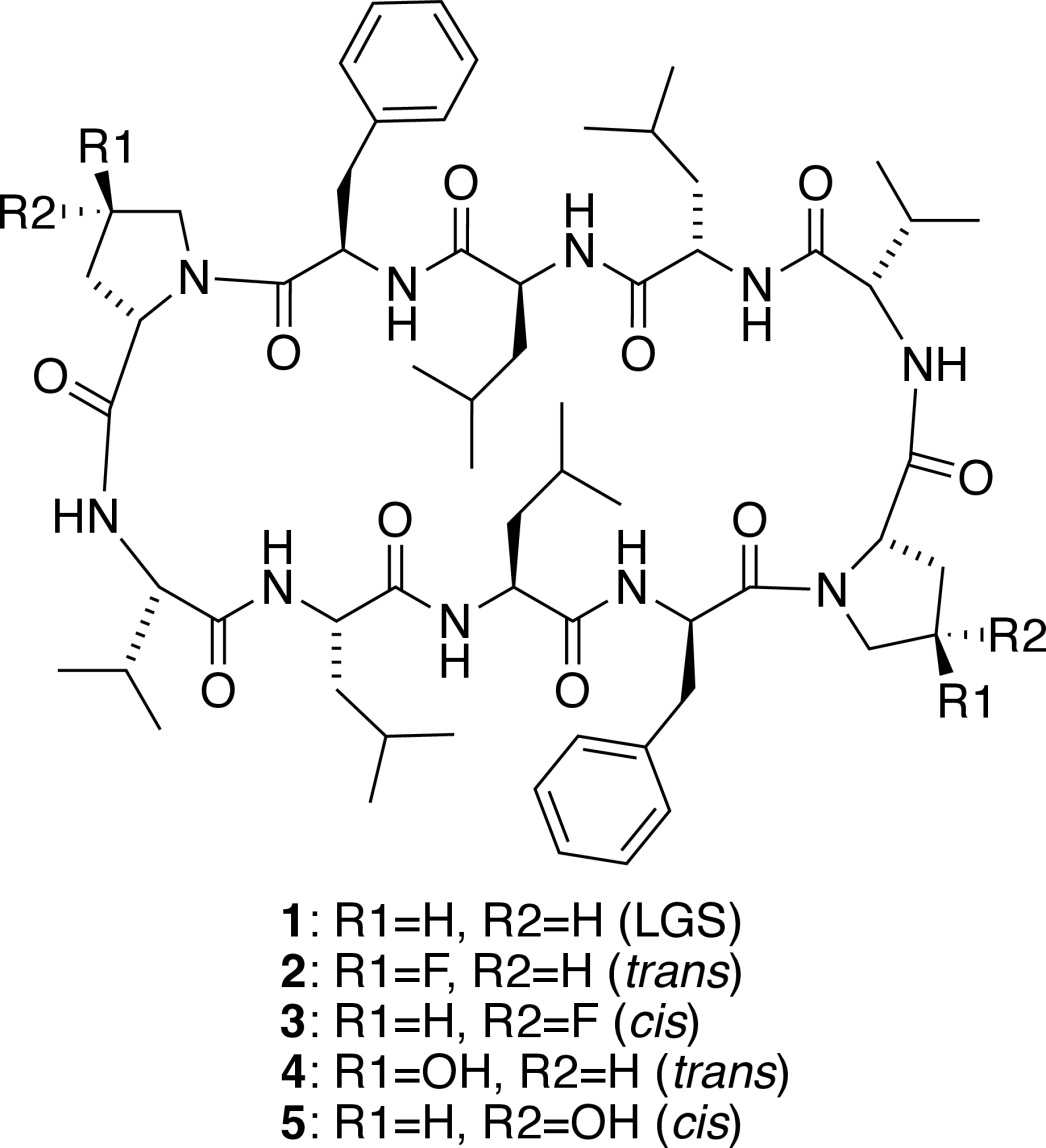


## 2. Structural commentary

**Figure 1:**
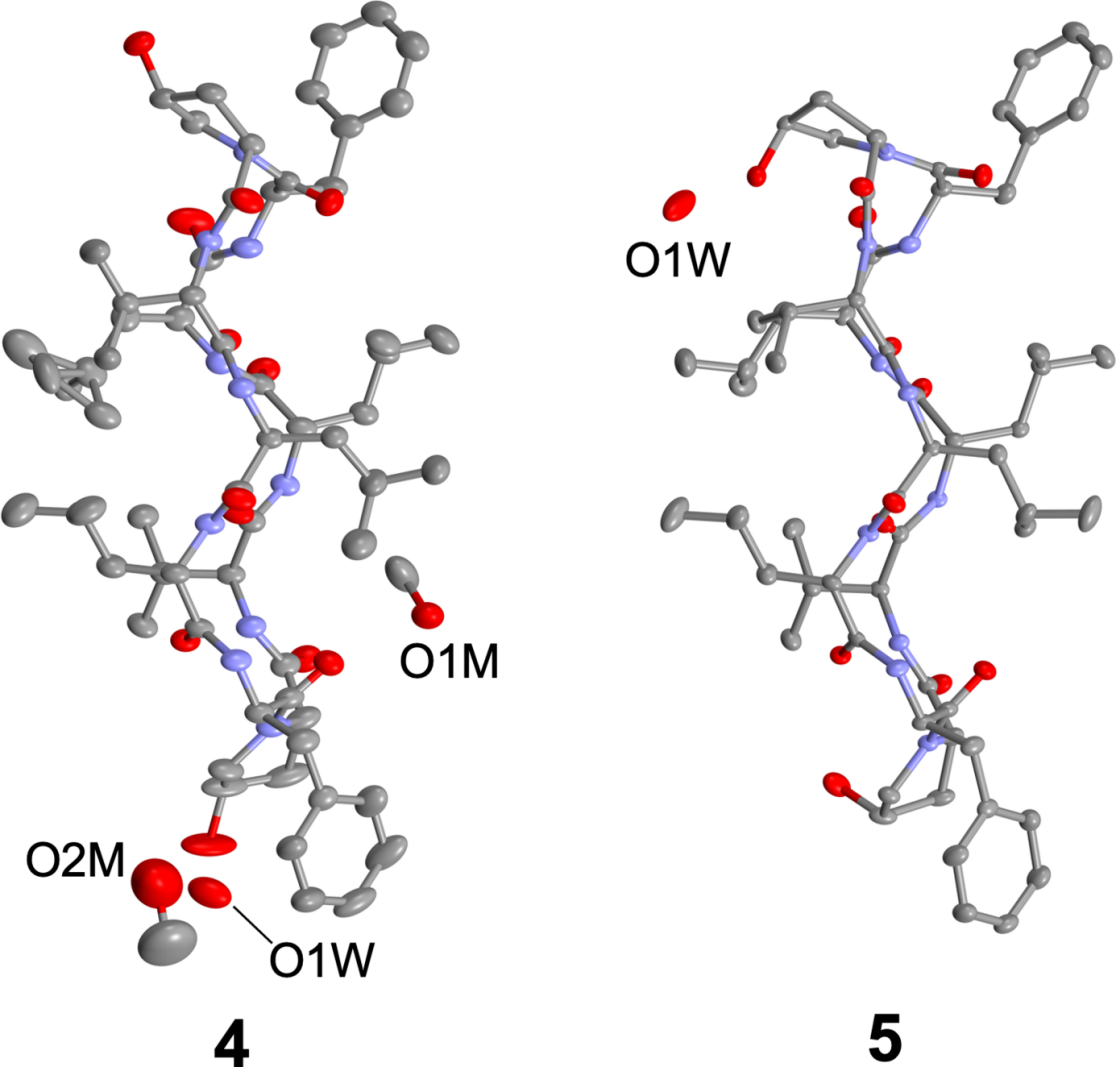
Mol­ecular structures of **4** and **5**. Hydrogen atoms are omitted for clarity. Oxygen atoms of solvent mol­ecules are labeled.

**Figure 2:**
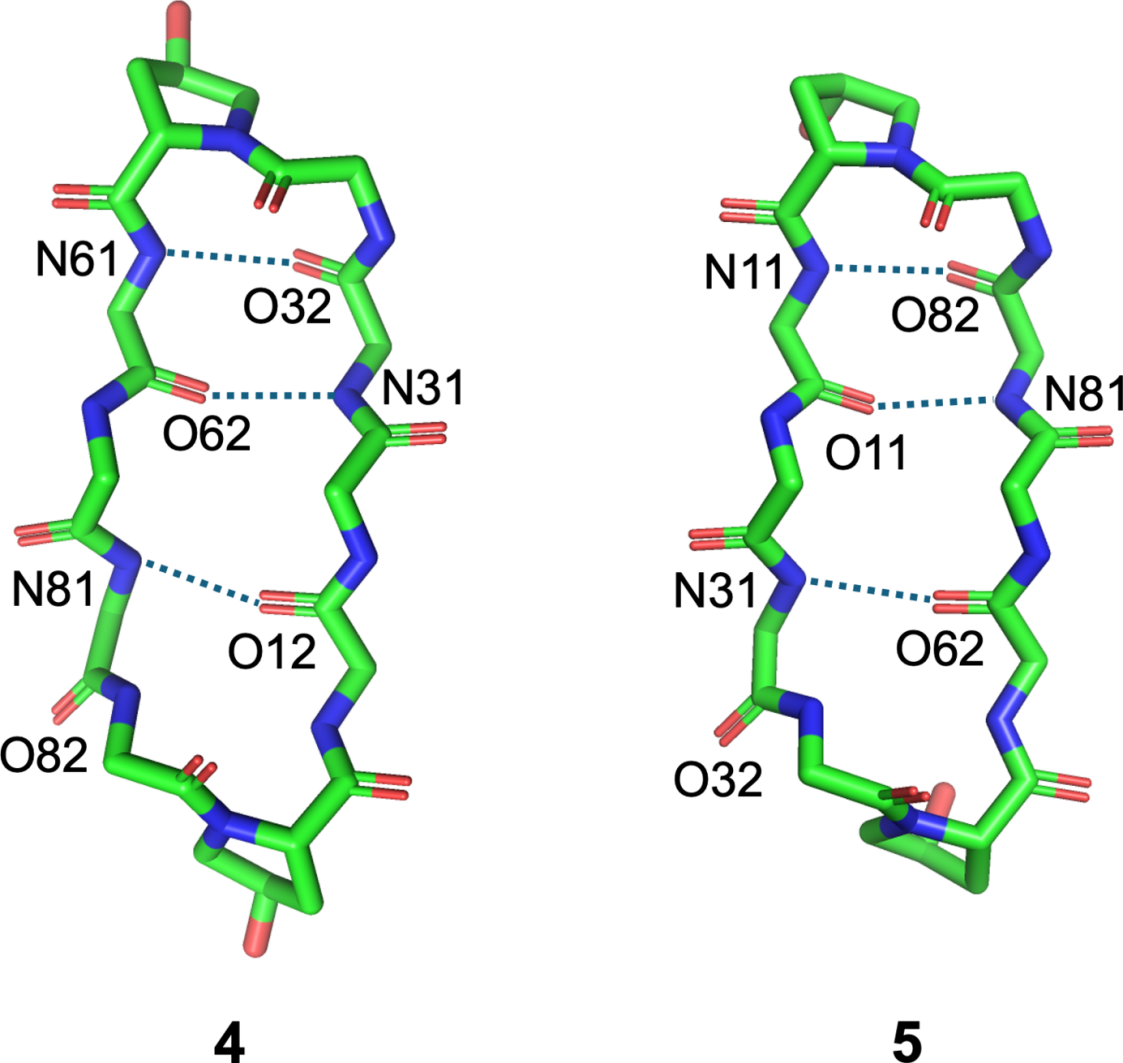
Backbone structures of **4** and **5**. Dotted lines show intra­molecular hydrogen bonds.

**Figure 3:**
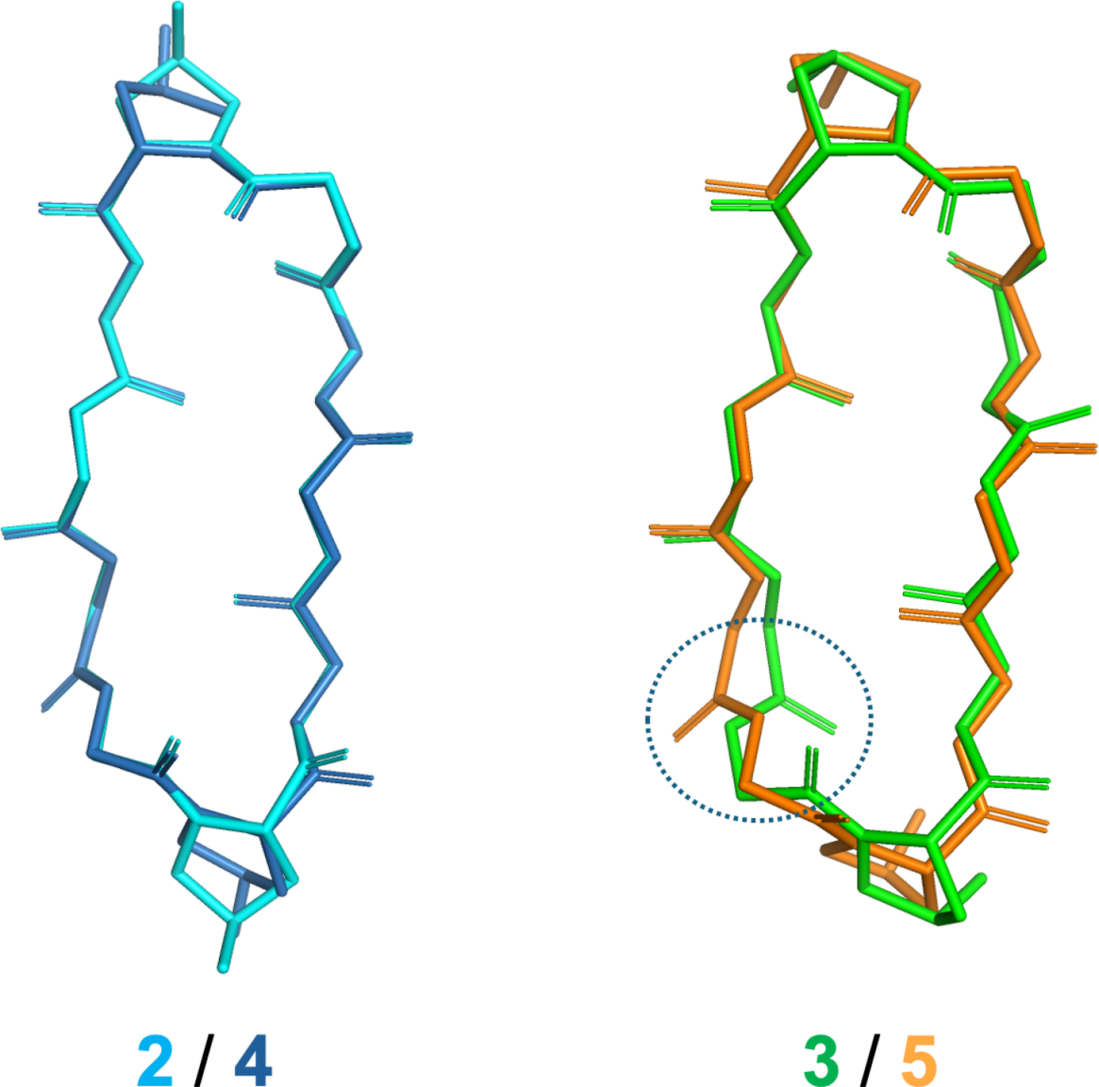
Superimposition of backbone structures. The circled area highlights a region with noticeable drift in the backbone structures Mol­ecular fittings were performed using *ProFit* (Martin, 2022 ▸) and gave the r.m.s. values of 0.099 and 0.498 Å for the **2**/**4** and **3**/**5** pairs, respectively.

Fig. 1 ▸ illustrates the structures of **4** and **5**, which have three intra­molecular hydrogen bonds (Table 1 ▸, Fig. 2 ▸) [**4**: N31⋯O62 = 2.838 (5), N61⋯O32 = 3.048 (5) and N81⋯O12 = 2.905 (4) Å, **5**: N11⋯O82 = 3.012 (2), N31⋯O62 = 2.985 (2) and N81⋯O12 = 2.831 (2) Å], forming a sheet structure. Additionally, two bends are observed (d-Phe-Pro moieties), one forming a β-turn of type II’, while the other is not classed into any β-turn (Table 2 ▸). Such asymmetrical structures arise from outward-facing carbonyl groups [O82 (**4**) and O32 (**5**)], preventing intra­molecular hydrogen-bond formation (Fig. 2 ▸). Comparing the peptide backbones, peptides **2** and **4**, with a 4-*trans*-substitution of Pro, exhibit similar structures with the asymmetric peptide backbone (Fig. 3 ▸). Conversely, peptides **3** and **5**, with *cis*-substitution, display differences in backbone structure attributed to the asymmetric structure of **5**.

The puckering parameters (Table 3 ▸) reveal that both peptides **4** and **5** exhibit Hyp in the down-puckering (Cβ-*exo*), which was somewhat unexpected. Table 4 ▸ outlines the puckering type and symmetry of the backbone structure. Peptides **1** and **3**, with the pyrrolidine ring in the down-puckering mode, exhibit symmetric backbone structures (peptide **1** contains three independent mol­ecules with coexisting structures), suggesting a preference for a symmetric backbone when the pyrrolidine ring is down-puckering. In contrast, peptide **2**, with tFPro-induced up-puckering, displays an asymmetric backbone structure. Inter­estingly, in peptide **4** tHyp remains in the down-puckering form, indicating that the 4-*trans*-hy­droxy group does not induce an up-puckering. Peptide **5**, also down-puckered, shows an asymmetric backbone. No clear relationship between Hyp puckering and backbone structure was observed in **4** and **5**.

**Figure 4:**
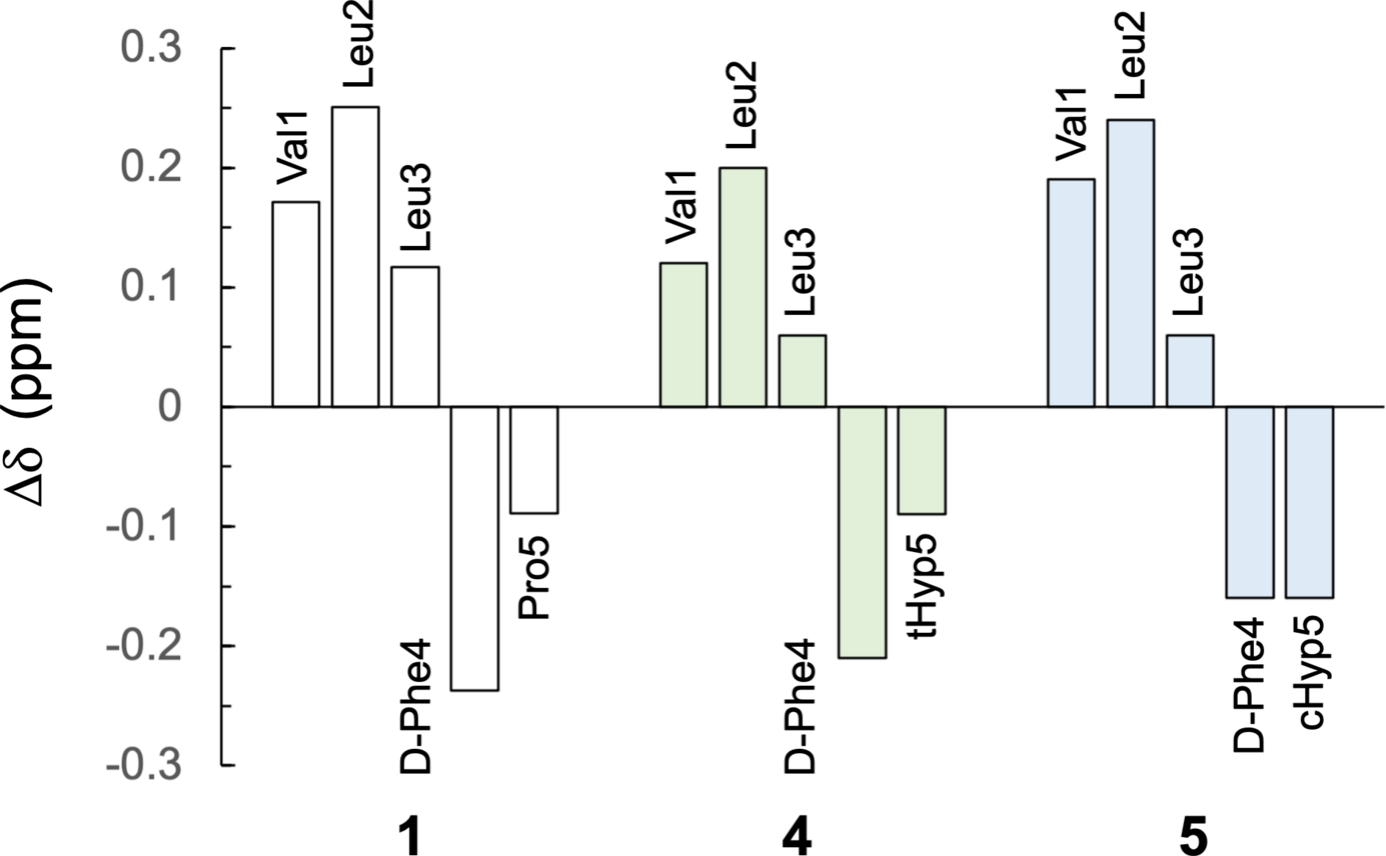
Plot of chemical shift perturbation (Δδ) of Hα atoms. The data of **1** are referenced from the previous report (Asano *et al.*, 2019 ▸).

**Figure 5:**
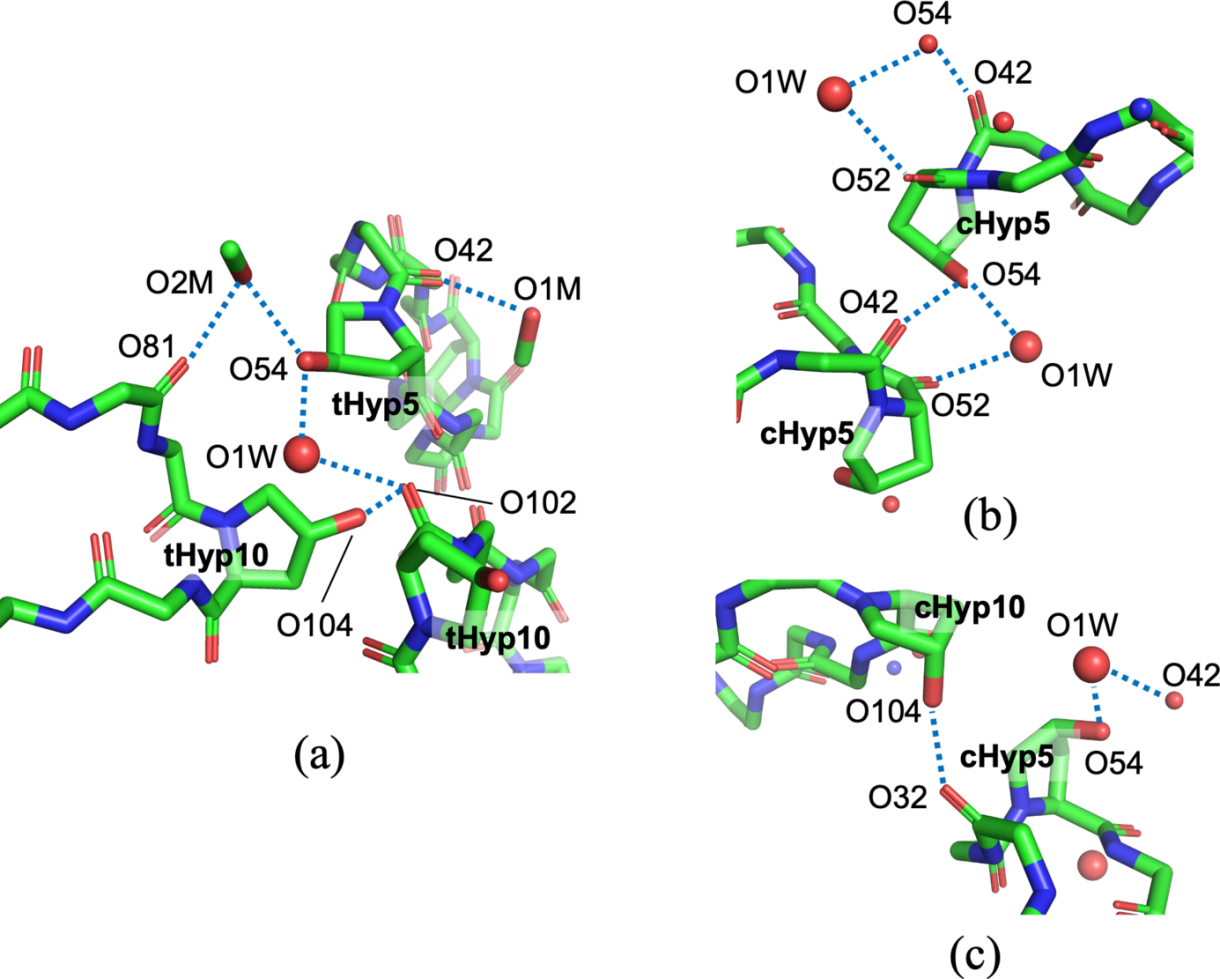
Hydrogen-bond networks relating to Hyp residues. (*a*) tHyp5 and tHyp10 of **4**. (*b*) cHyp5 and (*c*) cHyp10 of **5**.

Chemical shift perturbation analysis (Tamaki *et al.*, 2010 ▸) shows significant differences of Δδ in Hα of peptides **2** and **3** with fluoride-Pro (Asano *et al.*, 2019 ▸). In constrast, peptides **4** and **5**, containing Hyp, exhibit similar Δδ values to **1** (Fig. 4 ▸), suggesting minimal influence on the peptide backbone. This could be attributed to the 4-position substitution of Hyp, where hy­droxy groups (O54 and O104) form hydrogen bonds. While peptides **4** and **5** form hydrogen bonds with solvent and neighboring mol­ecules (Table 1 ▸), no notable differences attributable to *trans*/*cis* isomerism are observed (Fig. 5 ▸). As a hypothesis, compared to peptides **2** and **3**, the hydrogen bonds formed by the hy­droxy groups of Hyp may reduce structural stresses from the *trans*/*cis*-geometry of Hyp, explaining the results of chemical shift perturbation.

## 3. Synthesis and crystallization

Petides **4** and **5** were synthesized using the conventional liquid-phase method (Izumiya *et al.*, 1997 ▸). Crystals were grown from aqueous methanol solution. The NMR spectrum was measured in DMSO-*d*_6_ on a Varian Unity Inova 500 MHz. ^1^H NMR (p.p.m.): **4** δ 0.76 (*d*, *J* = 6.6 Hz,3*H*, Leu3-δ1CH_3_3), 0.78 (*d*, *J* = 6.6 Hz, Val1-γ1CH_3_), 0.78 (*d*, *J* = 6.6 Hz,3*H*, Leu3-δ2CH_3_3), 0.79 (*d*, *J* = 6.6 Hz, Val1-γ2CH_3_), 0.82 (*d*, *J* = 6.6 Hz, 3H, Leu2-δ1CH_3_), 0.82 (*d*, *J* = 6.6 Hz, 3H, Leu2-δ2CH_3_), 1.28 (*m*, *J* = 1.28 Hz, 1H, Leu2-βCH), 1.28 (*m*, 1H, Leu3-βCH), 1.41 (*m*, 1H, Leu3-β′CH), 1.41 (*m*, 1H, Leu3-γCH), 1.59 (non., *J* = 6.6 Hz, 1H, Leu2-γCH), 1.66 (*m*, 1H, tHyp5-βCH), 1.71 (*m*, *J* = 1.71 Hz, 1H, Leu2-β′CH), 2.03 (*m*, 2H, Val1-βCH), 2.04 (*m*, 1H, tHyp5-β′CH), 2.71 (*dd*, *J* = 9.9 Hz, 6.0 Hz, 1H, tHyp5-δCH), 2.83 (*dd*, *J* = 13.5 Hz, 7.2 Hz, 1H, D-Phe4-βCH), 2.90 (*dd*, *J* = 13.5 Hz, 7.2 Hz, 1H, D-Phe4-β′CH), 3.71 (*dd*, *J* = 9.9 Hz, 6.0 Hz, 1H, tHyp5-δ′CH), 4.13 (*m*, 1H, tHyp5-γCH), 4.30 (*dd*, *J* = 9.0 Hz, 6.6 Hz, 1H, Val1-αCH), 4.35 (*dd*, *J* = 8.4 Hz, 3.6 Hz, 1H, tHyp5-αCH), 4.44 (*q*, *J* = 8.4 Hz, 1H, Leu3-αCH), 4.45 (*q*, *J* = 7.2 Hz, 1H, D-Phe4-αCH), 4.58 (*q*, *J* = 7.8 Hz, 1H, Leu2-αCH), 5.12 (*d*, *J* = 3.6 Hz, 1H, tHyp5-γOH), 7.15 (*br*, 1H, Val1-CONH), 7.22 (*m*, 3H, D-Phe4-ArH), 7.28 (*m*, 2H, D-Phe4-ArH), 8.27 (*d*, *J* = 8.4 Hz, 1H, Leu3-CONH), 8.30 (*d*, *J* = 7.2 Hz, 1H, D-Phe4-CONH), 8.36 (*d*, *J* = 7.8 Hz, 1H, Leu2-CONH).

**5** δ 0.77 (*d*, *J* = 6.6 Hz, 3H, Leu3-δ1CH_3_), 0.78 (*d*, *J* = 6.6 Hz, 3H, Val1-γ1CH_3_), 0.79 (*d*, *J* = 6.6 Hz, 3H, Val1-γ2CH_3_), 0.80 (*d*, *J* = 6.6 Hz, 3H, Leu3-δ2CH_3_), 0.85 (*d*, *J* = 6.6 Hz, 3H, Leu2-δ1CH_3_), 0.86 (*d*, *J* = 6.6 Hz, 3H, Leu2-δ2CH_3_), 1.28 (*m*, 1H, Leu2-βCH), 1.36 (*m*, 1H, Leu3-βCH), 1.48 (*m*, 1H, Leu3-β′CH), 1.48 (*m*, 1H, Leu3-γCH), 1.60 (*m*, 1H, Leu2-γCH), 1.80 (*m*, 1H, Leu2-β′CH), 1.86 (*m*, 1H, cHyp5-βCH), 1.94 (*m*, 1H, cHyp5-β′CH), 1.97 (*m*, 1H, Val1-βCH), 2.79 (*dd*, *J* = 12.9 Hz, 6.0 Hz, 1H, D-Phe4-βCH), 2.89 (*dd*, *J* = 12.9 Hz, 8.4 Hz, 1H, D-Phe4-β′CH), 3.01 (*m*, 1H, cHyp5-δCH), 3.34 (*m*, 1H, cHyp5-δ′CH), 3.92 (*br*, 1H, cHyp5-γCH), 4.28 (*dd*, *J* = 9.6 Hz, 1.8 Hz, 1H, cHyp5-αCH), 4.37 (*dd*, *J* = 9.0 Hz, 6.6 Hz, 1H, Val1-αCH), 4.44 (*td*, *J* = 9.0 Hz, 6.6 Hz, 1H, Leu3-αCH), 4.50 (*td*, *J* = 8.4 Hz, 6.0 Hz, 1H, D-Phe4-αCH), 4.62 (*q*, *J* = 8.4 Hz, 1H, Leu2-αCH), 4.96 (*d*, *J* = 2.4 Hz, 1H, cHyp5-γOH), 6.98 (*d*, *J* = 9.0 Hz, 1H, Val1-CONH), 7.22 (*m*, 3H, D-Phe4-ArH), 7.23 (*m*, 2H, D-Phe4-ArH), 7.95 (*d*, *J* = 6.0 Hz, 1H, D-Phe4-CONH), 8.36 (*d*, *J* = 9.0 Hz, 1H, Leu3-CONH), 8.50 (*d*, *J* = 8.4 Hz, 1H, Leu2-CONH).

## 4. Refinement

Crystal data, data collection and structure refinement details are summarized in Table 5 ▸. All H atoms were located in difference maps and then treated as riding in geometrically idealized positions with constrained distances set to 0.93 Å (C*sp*^2^—H), 0.98 Å (*R*_3_—CH), 0.97 Å (*R*_2_—CH_2_), 0.96 Å (R—CH_3_), 082 Å (R—OH) and 0.86 Å (N*sp*^2^—H). *U*_iso_(H) parameters were set to values of either 1.2 or 1.5 (methyl and hy­droxy groups) times *U*_eq_ of the attached atom. The H atoms attached to water (**5**) and the hydroxyl groups of Hyp (**4** and **5**) were located in the Fourier map considering donor–acceptor pairs in the hydrogen-bond network, and included in the calculation of structure factors with fixed position. The H atoms attached to methanol and water molecules of (**4**) were located in the Fourier map and included in the refinement with restraints for the bond lengths and angles.

## Supplementary Material

Crystal structure: contains datablock(s) global, 4, 5. DOI: 10.1107/S2056989024007771/jp2008sup1.cif

Structure factors: contains datablock(s) 4. DOI: 10.1107/S2056989024007771/jp20084sup2.hkl

Structure factors: contains datablock(s) 5. DOI: 10.1107/S2056989024007771/jp20085sup3.hkl

CCDC references: 2376129, 2376128

Additional supporting information:  crystallographic information; 3D view; checkCIF report

## Figures and Tables

**Table 1:** Hydrogen-bonding geometry (Å, °)

*D*—H⋯*A*	H⋯*A*	*D*⋯*A*	*D*—H⋯*A*
**4**			
β-sheet			
N31—H31⋯O62	2.01	2.838 (5)	162
N61—H61⋯O32	2.22	3.048 (5)	160
N81—H81⋯O12	2.19	2.905 (4)	140
Inter­molecular			
N21—H21⋯O52^i^	2.25	3.090 (4)	165
N41—H41⋯O72^i^	2.13	2.966 (5)	165
N71—H71⋯O22^ii^	1.97	2.818 (4)	168
O104—H10*E*⋯O102^iii^	2.06	2.869 (5)	168
Solvents relating			
O54—H54⋯O2*M*	1.99	2.725 (9)	149
O1*W*—H2*W*⋯O54	2.01	2.847 (7)	163
O1*W*—H1*W*⋯O102^ii^	2.03	2.927 (6)	178
O1*M*—H1*M*⋯O42	2.03	2.831 (5)	166
O2*M*—H2*MA*⋯O82^iv^	1.99	2.727 (8)	144
			
**5**			
β-sheet			
N11—H11⋯O82	2.19	3.012 (2)	159
N31—H31⋯O62	2.19	2.985 (2)	155
N81—H81⋯O12	1.98	2.831 (2)	173
Inter­molecular			
N21—H21⋯O72^v^	2.03	2.870 (2)	164
O54—H54*A*⋯O42^vi^	1.94	2.759 (2)	177
N71—H71⋯O102^vii^	2.16	3.000 (2)	166
N91—H91⋯O22^vii^	2.13	2.949 (2)	159
O104—H10*C*⋯O32^viii^	2.01	2.823 (3)	170
Solvent relating			
O1*W*—H1⋯O54	2.03	2.870 (3)	163
O1*W*—H2⋯O52^vi^	2.12	2.950 (3)	148

Symmetry codes: (i) −*x* + 1; *y* + 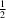, −*z* + 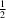; (ii) −*x* + 1, *y* − 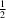, −*z* + 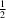; (iii) *x* + 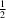, −*y* + 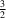, −*z* + 1; (iv) −*x* + 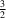, −*y* + 1, *z* − 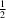; (v) −*x* + 1, *y* + 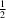, −*z* + 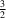; (vi) *x* + 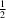, −*y* + 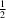, −*z* + 1; (vii) −*x* + 1, *y* − 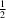, −*z* + 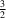; (viii) −*x* + 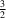, −*y* + 1, *z* + 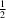.

**Table 2:** Selected torsion angles (°)

Residue	**4**		**5**	
	φ	ψ	φ	ψ
Val1	−120.8 (4)	142.0 (3)	−128.7 (2)	118.2 (2)
Leu2	−129.5 (4)	115.4 (4)	−99.6 (2)	117.0 (2)
Leu3	−120.3 (4)	97.3 (4)	−95.4 (2)	−30.0 (3)
D-Phe4	56.3 (5)^*a*^	−124.7 (4)^*a*^	−96.0 (2)	−71.0 (2)
t/cHyp5	−82.9 (5)^*a*^	9.2 (6)^*a*^	−79.5 (2)	−2.3 (3)
Val6	−119.4 (4)	120.1 (4)	−153.2 (2)	146.4 (2)
Leu7	−98.0 (4)	107.2 (4)	−122.5 (2)	121.6 (2)
Leu8	−80.4 (5)	−32.5 (5)	−122.3 (2)	91.1 (2)
D-Phe9	146.8 (4)	−92.4 (4)	54.1 (3)^*a*^	−124.2 (2)^*a*^
t/cHyp10	−77.2 (5)	−12.4 (5)	−82.0 (3)^*a*^	17.8 (3)^*a*^

Note: (*a*) The angles are classed to Type II’ β-turn, defined as (φ, ψ) = (60, −120)_*i* + 1_ and (−80, 0)_*i* + 2_.

**Table 3:** Puckering parameters (Å, °)

Residue	*Q*_2_^*a*^	φ_2_^*a*^	χ_1_	χ_2_	χ_3_	χ_4_	θ	Type
**4**								
tHyp5	0.398 (7)	81.8 (8)	35.8 (5)	−40.3 (5)	28.8 (5)	−6.4 (5)	−18.2 (5)	down
tHyp10	0.335 (5)	81.1 (7)	30.5 (4)	−34.0 (4)	24.1 (4)	−5.1 (4)	−16.0 (4)	down
**5**								
cHyp5	0.369 (2)	84.1 (3)	32.5 (2)	−38.0 (2)	28.1 (2)	−7.7 (2)	−15.5 (2)	down
cHyp10	0.379 (3)	85.7 (3)	33.0 (2)	−39.4 (2)	29.7 (2)	−9.0 (2)	−15.0 (2)	down

Note: (*a*) Defined by Cremer & Pople (1975 ▸). Puckering parameters were calculated using *PLATON* (Spek, 2020 ▸).

**Table 4:** The puckering of the pyrrolidine ring and symmetry of the peptide backbone

Compound	Puckering	Structure^*a*^	Compound	Puckering	Structure^*a*^
**1**	down	s+a			
**2**	up	a	**4**	down	a
**3**	down	s	**5**	down	a

Note: (*a*) The letters ‘a’ and ‘s’ indicate the asymmetric and symmetric peptide rings, respectively.

**Table 5:** Experimental details

	**4**	**5**
Crystal data		
Chemical formula	C_62_H_94_N_10_O_12_·2CH_4_O·H_2_O	C_62_H_94_N_10_O_12_·H_2_O
*M*_r_	1253.57	1189.48
Crystal system, space group	Orthorhombic, *P*2_1_2_1_2_1_	Orthorhombic, *P*2_1_2_1_2_1_
Temperature (K)	100	100
*a*, *b*, *c* (Å)	11.8909 (1), 18.8562 (2), 30.3163 (3)	12.0818 (1), 19.0030 (2), 28.9218 (2)
*V* (Å^3^)	6797.4 (1)	6640.17 (10)
*Z*	4	4
Radiation type	Cu *K*α	Cu *K*α
μ (mm^−1^)	0.71	0.68
Crystal size (mm)	0.45 × 0.16 × 0.10	0.40 × 0.10 × 0.05

Data collection		
Diffractometer	RIGAKU XtaLAB P200K	RIGAKU Xtalab P200K
Absorption correction	Multi-scan (*CrysAlis PRO*; Rigaku OD, 2015 ▸)	Multi-scan (*CrysAlis PRO*; Rigaku OD, 2015 ▸)
*T*_min_, *T*_max_	0.618, 1.0	0.830, 1.000
No. of measured, independent and observed [*I* > 2σ(*I*)] reflections	42425, 12349, 11935	31704, 9942, 9728
*R*_int_	0.026	0.023
θ_max_ (°)	68.3	60.7
(sin θ/λ)_max_ (Å^−1^)	0.602	0.566

Refinement		
*R*[*F*^2^ > 2σ(*F*^2^)], *wR*(*F*^2^), *S*	0.067, 0.203, 1.09	0.030, 0.082, 1.04
No. of reflections	12349	9942
No. of parameters	835	768
No. of restraints	29	3
H-atom treatment	H atoms treated by a mixture of independent and constrained refinement	H-atom parameters constrained
Δρ_max_, Δρ_min_ (e Å^−3^)	0.98, −1.30	0.84, −0.21
Absolute structure	Flack *x* determined using 5149 quotients [(*I*^+^)−(*I*^−^)]/[(*I*^+^)+(*I*^−^)] (Parsons et al., 2013 ▸)	Flack *x* determined using 4245 quotients [(*I*^+^)−(*I*^−^)]/[(*I*^+^)+(*I*^−^)] (Parsons et al., 2013 ▸)
Absolute structure parameter	0.08 (4)	0.00 (3)

Computer programs: *CrysAlis PRO* (Rigaku OD, 2015 ▸), *SHELXT* (Sheldrick, 2015*a* ▸) and *SHELXL2017/1* (Sheldrick, 2015*b* ▸).

## supplementary crystallographic information

### cyclo(Val-Leu-Leu-D-Phe-tHyp)2 methanol disolvate monohydrate (4) Crystal data

**Table:**

C_62_H_94_N_10_O_12_·2CH_4_O·H_2_O	*D*_x_ = 1.225 Mg m^−^^3^
*M**_r_* = 1253.57	Cu *K*α radiation, λ = 1.54184 Å
Orthorhombic, *P*2_1_2_1_2_1_	Cell parameters from 27488 reflections
*a* = 11.8909 (1) Å	θ = 2.8–70.2°
*b* = 18.8562 (2) Å	µ = 0.71 mm^−^^1^
*c* = 30.3163 (3) Å	*T* = 100 K
*V* = 6797.4 (1) Å^3^	Needle, colourless
*Z* = 4	0.45 × 0.16 × 0.10 mm
*F*(000) = 2712	

### cyclo(Val-Leu-Leu-D-Phe-tHyp)2 methanol disolvate monohydrate (4) Data collection

**Table:**

RIGAKU XtaLAB P200K diffractometer	12349 independent reflections
Radiation source: rotating anode	11935 reflections with *I* > 2σ(*I*)
Detector resolution: 5.8140 pixels mm^-1^	*R*_int_ = 0.026
ω–scan	θ_max_ = 68.3°, θ_min_ = 2.9°
Absorption correction: multi-scan (CrysAlisPro; Rigaku OD, 2015)	*h* = −14→14
*T*_min_ = 0.618, *T*_max_ = 1.0	*k* = −17→22
42425 measured reflections	*l* = −35→36

### cyclo(Val-Leu-Leu-D-Phe-tHyp)2 methanol disolvate monohydrate (4) Refinement

**Table:**

Refinement on *F*^2^	Hydrogen site location: mixed
Least-squares matrix: full	H atoms treated by a mixture of independent and constrained refinement
*R*[*F*^2^ > 2σ(*F*^2^)] = 0.067	*w* = 1/[σ^2^(*F*_o_^2^) + (0.1438*P*)^2^ + 2.8253*P*] where *P* = (*F*_o_^2^ + 2*F*_c_^2^)/3
*wR*(*F*^2^) = 0.203	(Δ/σ)_max_ = 0.011
*S* = 1.09	Δρ_max_ = 0.98 e Å^−^^3^
12349 reflections	Δρ_min_ = −1.30 e Å^−^^3^
835 parameters	Absolute structure: Flack *x* determined using 5149 quotients [(*I*^+^)-(*I*^-^)]/[(*I*^+^)+(*I*^-^)] (Parsons et al., 2013)
29 restraints	Absolute structure parameter: 0.08 (4)

### cyclo(Val-Leu-Leu-D-Phe-tHyp)2 methanol disolvate monohydrate (4) Special details

**Table:**

Geometry. All esds (except the esd in the dihedral angle between two l.s. planes) are estimated using the full covariance matrix. The cell esds are taken into account individually in the estimation of esds in distances, angles and torsion angles; correlations between esds in cell parameters are only used when they are defined by crystal symmetry. An approximate (isotropic) treatment of cell esds is used for estimating esds involving l.s. planes.

### cyclo(Val-Leu-Leu-D-Phe-tHyp)2 methanol disolvate monohydrate (4) Fractional atomic coordinates and isotropic or equivalent isotropic displacement parameters (Å^2^)

**Table:**

	*x*	*y*	*z*	*U*_iso_*/*U*_eq_	Occ. (<1)
N11	0.7511 (3)	0.74028 (18)	0.43846 (10)	0.0302 (7)	
H11	0.770195	0.696322	0.439474	0.036*	
C11	0.7533 (3)	0.7758 (2)	0.39577 (12)	0.0304 (8)	
H11A	0.727017	0.824775	0.399246	0.037*	
C12	0.6729 (3)	0.7356 (2)	0.36512 (12)	0.0277 (7)	
O12	0.6704 (3)	0.67041 (15)	0.36572 (10)	0.0356 (6)	
C13	0.8739 (4)	0.7762 (2)	0.37671 (13)	0.0347 (8)	
H13	0.895444	0.727050	0.370346	0.042*	
C14	0.9574 (5)	0.8064 (4)	0.40964 (18)	0.0581 (14)	
H14A	0.953849	0.779726	0.436570	0.087*	
H14B	1.031958	0.803513	0.397628	0.087*	
H14C	0.939160	0.855093	0.415515	0.087*	
C15	0.8788 (4)	0.8180 (4)	0.33395 (16)	0.0528 (13)	
H15A	0.825827	0.798624	0.313307	0.079*	
H15B	0.860409	0.866711	0.339699	0.079*	
H15C	0.953207	0.815132	0.321812	0.079*	
N21	0.6130 (3)	0.77560 (17)	0.33758 (11)	0.0291 (7)	
H21	0.620698	0.820899	0.338867	0.035*	
C21	0.5345 (3)	0.7459 (2)	0.30511 (12)	0.0311 (8)	
H21A	0.544977	0.694467	0.303064	0.037*	
C22	0.5655 (4)	0.7805 (2)	0.26146 (13)	0.0319 (8)	
O22	0.5585 (3)	0.84551 (15)	0.25738 (9)	0.0392 (7)	
C23	0.4142 (4)	0.7625 (2)	0.31965 (14)	0.0347 (8)	
H23A	0.405677	0.747000	0.349983	0.042*	
H23B	0.404608	0.813550	0.319307	0.042*	
C24	0.3186 (4)	0.7296 (3)	0.29258 (18)	0.0519 (12)	
H24	0.327963	0.678135	0.295158	0.062*	
C25	0.3136 (6)	0.7446 (4)	0.2449 (2)	0.0689 (17)	
H25A	0.384700	0.733302	0.231625	0.103*	
H25B	0.255624	0.716374	0.231613	0.103*	
H25C	0.297362	0.793960	0.240403	0.103*	
C26	0.2075 (4)	0.7467 (4)	0.3152 (2)	0.0567 (13)	
H26A	0.213421	0.736402	0.346154	0.085*	
H26B	0.190369	0.796084	0.311307	0.085*	
H26C	0.148631	0.718498	0.302517	0.085*	
N31	0.6051 (3)	0.73732 (18)	0.22955 (11)	0.0326 (7)	
H31	0.608008	0.692423	0.234401	0.039*	
C31	0.6433 (4)	0.7647 (2)	0.18698 (13)	0.0336 (8)	
H31A	0.633395	0.816270	0.186285	0.040*	
C32	0.5716 (4)	0.7308 (2)	0.15055 (12)	0.0325 (8)	
O32	0.5943 (3)	0.67093 (15)	0.13636 (9)	0.0362 (6)	
C33	0.7670 (4)	0.7467 (3)	0.17898 (15)	0.0431 (10)	
H33A	0.774911	0.695502	0.178268	0.052*	
H33B	0.788401	0.764618	0.150195	0.052*	
C34	0.8490 (4)	0.7765 (3)	0.21344 (17)	0.0487 (11)	
H34	0.830609	0.754519	0.241837	0.058*	
C35	0.8372 (6)	0.8560 (3)	0.2189 (2)	0.0605 (15)	
H35A	0.760630	0.867353	0.226147	0.091*	
H35B	0.885710	0.871854	0.242249	0.091*	
H35C	0.857672	0.879200	0.191929	0.091*	
C36	0.9683 (6)	0.7559 (4)	0.2020 (3)	0.0719 (18)	
H36A	0.973086	0.705394	0.198788	0.108*	
H36B	0.989868	0.778367	0.174902	0.108*	
H36C	1.017906	0.771021	0.225222	0.108*	
N41	0.4847 (3)	0.76819 (18)	0.13525 (11)	0.0358 (7)	
H41	0.469741	0.809393	0.145909	0.043*	
C41	0.4153 (4)	0.7382 (2)	0.10018 (13)	0.0349 (9)	
H41A	0.461268	0.731981	0.073628	0.042*	
C42	0.3692 (4)	0.6662 (2)	0.11459 (14)	0.0347 (9)	
O42	0.3136 (3)	0.66123 (17)	0.14827 (10)	0.0418 (7)	
C43	0.3166 (5)	0.7876 (2)	0.08969 (16)	0.0437 (10)	
H43A	0.267756	0.791326	0.115191	0.052*	
H43B	0.344667	0.834569	0.082723	0.052*	
C44	0.2511 (4)	0.7585 (2)	0.05083 (15)	0.0373 (9)	
C45	0.1610 (5)	0.7130 (3)	0.0566 (2)	0.0599 (15)	
H45	0.136209	0.702328	0.084932	0.072*	
C46	0.1072 (6)	0.6832 (3)	0.0204 (3)	0.074 (2)	
H46	0.047616	0.651954	0.024686	0.089*	
C47	0.1409 (6)	0.6992 (3)	−0.0216 (2)	0.0677 (18)	
H47	0.104542	0.679259	−0.045772	0.081*	
C48	0.2280 (5)	0.7446 (4)	−0.0275 (2)	0.0676 (17)	
H48	0.250581	0.756320	−0.055994	0.081*	
C49	0.2840 (4)	0.7740 (3)	0.00839 (18)	0.0515 (12)	
H49	0.344388	0.804442	0.003661	0.062*	
N51	0.3898 (3)	0.61001 (19)	0.08838 (12)	0.0376 (8)	
C51	0.3384 (5)	0.5413 (2)	0.09743 (17)	0.0466 (12)	
H51	0.260115	0.548211	0.106586	0.056*	
C52	0.4006 (4)	0.4963 (2)	0.13229 (15)	0.0372 (9)	
O52	0.3694 (3)	0.43453 (16)	0.13855 (11)	0.0439 (7)	
C53	0.3413 (6)	0.5063 (3)	0.0515 (2)	0.0655 (19)	
H53A	0.277608	0.521094	0.033723	0.079*	
H53B	0.341028	0.454963	0.053849	0.079*	
C54	0.4513 (7)	0.5328 (3)	0.03184 (17)	0.0633 (18)	
H54A	0.514654	0.506975	0.044871	0.076*	
C55	0.4539 (5)	0.6105 (3)	0.04670 (14)	0.0471 (11)	
H55A	0.530541	0.626446	0.051475	0.057*	
H55B	0.418451	0.641031	0.025023	0.057*	
O54	0.4519 (6)	0.5242 (3)	−0.01478 (13)	0.0907 (19)	
H54	0.447565	0.563198	−0.026631	0.136*	
N61	0.4835 (3)	0.52818 (18)	0.15465 (11)	0.0343 (7)	
H61	0.497931	0.572021	0.149251	0.041*	
C61	0.5502 (4)	0.4912 (2)	0.18787 (13)	0.0328 (8)	
H61A	0.523587	0.442168	0.190241	0.039*	
C62	0.5362 (4)	0.5279 (2)	0.23276 (13)	0.0325 (8)	
O62	0.5664 (3)	0.58954 (16)	0.23884 (10)	0.0428 (7)	
C63	0.6751 (4)	0.4907 (2)	0.17533 (13)	0.0352 (9)	
H63	0.700105	0.539978	0.172186	0.042*	
C64	0.6915 (4)	0.4534 (2)	0.13108 (14)	0.0408 (10)	
H64A	0.646433	0.476384	0.109026	0.061*	
H64B	0.769302	0.455817	0.122730	0.061*	
H64C	0.669128	0.404709	0.133670	0.061*	
C65	0.7458 (4)	0.4557 (3)	0.21156 (15)	0.0430 (10)	
H65A	0.733900	0.480106	0.238964	0.064*	
H65B	0.723724	0.406982	0.214685	0.064*	
H65C	0.823898	0.458091	0.203744	0.064*	
N71	0.4937 (3)	0.48684 (17)	0.26479 (11)	0.0325 (7)	
H71	0.467375	0.445760	0.258034	0.039*	
C71	0.4906 (4)	0.5099 (2)	0.31112 (13)	0.0341 (9)	
H71A	0.496598	0.561724	0.312559	0.041*	
C72	0.5925 (4)	0.4763 (2)	0.33371 (13)	0.0345 (9)	
O72	0.5959 (3)	0.41255 (15)	0.34212 (11)	0.0410 (7)	
C73	0.3817 (4)	0.4860 (2)	0.33336 (14)	0.0370 (9)	
H73A	0.373229	0.435367	0.329003	0.044*	
H73B	0.319000	0.509217	0.318853	0.044*	
C74	0.3753 (5)	0.5018 (2)	0.38287 (14)	0.0419 (10)	
H74	0.442177	0.480912	0.396632	0.050*	
C75	0.3773 (5)	0.5818 (3)	0.39270 (16)	0.0505 (12)	
H75A	0.442731	0.602718	0.379480	0.076*	
H75B	0.310938	0.603580	0.380723	0.076*	
H75C	0.379298	0.589191	0.424024	0.076*	
C76	0.2738 (6)	0.4663 (3)	0.40298 (18)	0.0594 (14)	
H76A	0.270933	0.476616	0.433963	0.089*	
H76B	0.206822	0.483728	0.388996	0.089*	
H76C	0.278949	0.415929	0.398759	0.089*	
N81	0.6768 (3)	0.52060 (18)	0.34352 (12)	0.0350 (7)	
H81	0.671795	0.564481	0.336048	0.042*	
C81	0.7769 (4)	0.4959 (2)	0.36664 (15)	0.0385 (9)	
H81A	0.796621	0.448728	0.355463	0.046*	
C82	0.7559 (5)	0.4904 (2)	0.41640 (16)	0.0439 (10)	
O82	0.8070 (5)	0.4471 (2)	0.43878 (13)	0.0692 (13)	
C83	0.8767 (4)	0.5462 (2)	0.35831 (16)	0.0439 (10)	
H83A	0.939835	0.530673	0.376124	0.053*	
H83B	0.855992	0.593370	0.368221	0.053*	
C84	0.9147 (5)	0.5508 (3)	0.31051 (18)	0.0526 (12)	
H84	0.847292	0.563022	0.293501	0.063*	
C85	0.9526 (7)	0.4796 (4)	0.2934 (3)	0.047 (2)	0.645 (14)
H85A	0.895937	0.444747	0.299512	0.070*	0.645 (14)
H85B	0.964521	0.482527	0.262120	0.070*	0.645 (14)
H85C	1.021559	0.466328	0.307639	0.070*	0.645 (14)
C86	0.9937 (9)	0.6087 (6)	0.3032 (4)	0.065 (3)	0.645 (14)
H86A	0.963114	0.651767	0.315064	0.098*	0.645 (14)
H86B	1.063520	0.598053	0.317685	0.098*	0.645 (14)
H86C	1.006483	0.614253	0.272166	0.098*	0.645 (14)
C85'	0.8673 (13)	0.6032 (7)	0.2834 (4)	0.055 (4)	0.355 (14)
H85D	0.898976	0.599805	0.254358	0.082*	0.355 (14)
H85E	0.787422	0.596470	0.281839	0.082*	0.355 (14)
H85F	0.883246	0.649177	0.295503	0.082*	0.355 (14)
C86'	1.0482 (11)	0.5716 (13)	0.3153 (6)	0.094 (8)	0.355 (14)
H86D	1.085100	0.537293	0.333707	0.141*	0.355 (14)
H86E	1.082614	0.571927	0.286707	0.141*	0.355 (14)
H86F	1.054842	0.617770	0.328448	0.141*	0.355 (14)
N91	0.6846 (4)	0.53721 (19)	0.43392 (12)	0.0392 (8)	
H91	0.652936	0.568062	0.417132	0.047*	
C91	0.6593 (4)	0.5371 (2)	0.48114 (14)	0.0392 (9)	
H91A	0.725601	0.519874	0.497087	0.047*	
C92	0.6395 (4)	0.6144 (2)	0.49370 (13)	0.0345 (9)	
O92	0.5478 (3)	0.64221 (18)	0.48768 (12)	0.0445 (7)	
C93	0.5593 (5)	0.4893 (3)	0.49263 (16)	0.0487 (12)	
H93A	0.489674	0.513451	0.485393	0.058*	
H93B	0.562995	0.446141	0.475239	0.058*	
C94	0.5600 (4)	0.4707 (2)	0.54107 (16)	0.0438 (11)	
C95	0.6451 (5)	0.4265 (3)	0.55792 (16)	0.0469 (11)	
H95	0.702407	0.410892	0.539459	0.056*	
C96	0.6441 (5)	0.4061 (3)	0.60210 (17)	0.0532 (13)	
H96	0.700168	0.376530	0.613024	0.064*	
C97	0.5605 (5)	0.4296 (3)	0.62927 (17)	0.0535 (13)	
H97	0.558991	0.415040	0.658574	0.064*	
C98	0.4784 (5)	0.4746 (3)	0.61374 (19)	0.0574 (13)	
H98	0.423205	0.491461	0.632779	0.069*	
C99	0.4778 (5)	0.4950 (3)	0.56943 (18)	0.0507 (12)	
H99	0.421841	0.525040	0.559008	0.061*	
N101	0.7299 (3)	0.65021 (18)	0.50826 (11)	0.0305 (7)	
C101	0.7206 (4)	0.7262 (2)	0.51769 (13)	0.0313 (8)	
H101	0.651237	0.735061	0.534236	0.038*	
C102	0.7212 (3)	0.7720 (2)	0.47610 (13)	0.0305 (8)	
O102	0.6959 (3)	0.83529 (16)	0.47898 (10)	0.0434 (7)	
C103	0.8221 (4)	0.7408 (2)	0.54753 (14)	0.0394 (9)	
H10A	0.848867	0.789052	0.543741	0.047*	
H10B	0.802988	0.733490	0.578295	0.047*	
C104	0.9107 (4)	0.6873 (2)	0.53232 (14)	0.0366 (9)	
H104	0.951983	0.705914	0.506848	0.044*	
C105	0.8423 (4)	0.6221 (2)	0.51895 (15)	0.0376 (9)	
H10C	0.875455	0.598960	0.493510	0.045*	
H10D	0.838119	0.588309	0.543027	0.045*	
O104	0.9860 (3)	0.6724 (2)	0.56743 (11)	0.0477 (8)	
H10E	1.049697	0.667366	0.557602	0.072*	
O1M	0.1547 (3)	0.5876 (2)	0.20060 (12)	0.0569 (9)	
H1M	0.191779	0.609604	0.182352	0.085*	
C1M	0.2259 (7)	0.5417 (5)	0.2245 (3)	0.086 (3)	
H1M1	0.273266	0.569033	0.243693	0.130*	
H1M2	0.271732	0.515138	0.204323	0.130*	
H1M3	0.181168	0.509626	0.241746	0.130*	
O1W	0.4313 (5)	0.3882 (2)	−0.05479 (14)	0.0692 (12)	
H1W	0.392 (7)	0.371 (4)	−0.032 (2)	0.104*	
H2W	0.450 (8)	0.430 (3)	−0.046 (3)	0.104*	
O2M	0.5246 (6)	0.6493 (3)	−0.0496 (3)	0.228 (7)	
H2MA	0.578 (5)	0.622 (4)	−0.041 (3)	0.342*	
C2M	0.4742 (16)	0.6599 (11)	−0.0908 (5)	0.180 (7)	
H2M1	0.459625	0.709557	−0.094945	0.269*	
H2M2	0.404764	0.634091	−0.092165	0.269*	
H2M3	0.523832	0.643424	−0.113595	0.269*	

### cyclo(Val-Leu-Leu-D-Phe-tHyp)2 methanol disolvate monohydrate (4) Atomic displacement parameters (Å^2^)

**Table:**

	*U*^11^	*U*^22^	*U*^33^	*U*^12^	*U*^13^	*U*^23^
N11	0.0426 (17)	0.0241 (16)	0.0240 (15)	0.0027 (13)	−0.0018 (13)	0.0007 (12)
C11	0.042 (2)	0.0244 (19)	0.0246 (18)	0.0025 (15)	−0.0047 (15)	0.0004 (14)
C12	0.0375 (18)	0.0214 (18)	0.0244 (17)	0.0016 (14)	0.0009 (14)	−0.0013 (13)
O12	0.0504 (16)	0.0219 (14)	0.0346 (14)	0.0025 (12)	−0.0073 (12)	0.0015 (11)
C13	0.042 (2)	0.032 (2)	0.0302 (19)	0.0000 (17)	−0.0027 (16)	0.0014 (15)
C14	0.052 (3)	0.082 (4)	0.040 (3)	−0.020 (3)	−0.008 (2)	0.009 (3)
C15	0.044 (2)	0.081 (4)	0.034 (2)	−0.006 (2)	−0.0015 (19)	0.012 (2)
N21	0.0426 (17)	0.0180 (14)	0.0268 (15)	−0.0015 (13)	−0.0038 (13)	−0.0011 (12)
C21	0.043 (2)	0.0232 (18)	0.0270 (18)	−0.0008 (15)	−0.0060 (15)	−0.0040 (15)
C22	0.045 (2)	0.024 (2)	0.0268 (18)	0.0035 (16)	−0.0063 (16)	−0.0014 (15)
O22	0.069 (2)	0.0182 (14)	0.0309 (14)	0.0048 (13)	−0.0030 (13)	−0.0009 (11)
C23	0.043 (2)	0.030 (2)	0.0319 (19)	−0.0010 (16)	−0.0040 (16)	−0.0032 (15)
C24	0.045 (2)	0.065 (3)	0.046 (3)	−0.009 (2)	−0.006 (2)	−0.014 (2)
C25	0.068 (4)	0.078 (5)	0.062 (4)	−0.001 (3)	−0.009 (3)	−0.015 (3)
C26	0.046 (3)	0.071 (4)	0.053 (3)	−0.012 (2)	−0.003 (2)	−0.008 (3)
N31	0.0506 (19)	0.0207 (15)	0.0266 (16)	0.0034 (13)	−0.0054 (14)	−0.0007 (12)
C31	0.051 (2)	0.0238 (19)	0.0258 (18)	−0.0001 (16)	−0.0056 (17)	−0.0012 (14)
C32	0.052 (2)	0.0229 (18)	0.0227 (17)	0.0001 (16)	−0.0022 (16)	−0.0006 (14)
O32	0.0505 (16)	0.0271 (14)	0.0311 (13)	0.0053 (12)	−0.0046 (12)	−0.0060 (11)
C33	0.057 (3)	0.040 (2)	0.032 (2)	0.000 (2)	−0.0020 (19)	−0.0063 (18)
C34	0.055 (3)	0.047 (3)	0.044 (2)	0.002 (2)	−0.013 (2)	−0.005 (2)
C35	0.070 (3)	0.050 (3)	0.061 (3)	−0.004 (3)	−0.025 (3)	−0.010 (2)
C36	0.068 (4)	0.068 (4)	0.080 (4)	0.002 (3)	−0.023 (3)	−0.014 (3)
N41	0.054 (2)	0.0233 (16)	0.0300 (16)	0.0039 (15)	−0.0064 (15)	−0.0065 (13)
C41	0.050 (2)	0.026 (2)	0.0287 (19)	0.0030 (17)	−0.0061 (16)	0.0012 (15)
C42	0.046 (2)	0.026 (2)	0.033 (2)	0.0050 (16)	−0.0100 (17)	−0.0034 (15)
O42	0.0553 (18)	0.0344 (16)	0.0358 (16)	−0.0016 (14)	0.0008 (14)	−0.0010 (12)
C43	0.060 (3)	0.028 (2)	0.043 (2)	0.0069 (19)	−0.011 (2)	−0.0025 (17)
C44	0.046 (2)	0.0238 (19)	0.042 (2)	0.0040 (16)	−0.0092 (18)	0.0040 (16)
C45	0.058 (3)	0.053 (3)	0.069 (3)	−0.015 (2)	−0.013 (3)	0.030 (3)
C46	0.078 (4)	0.040 (3)	0.103 (5)	−0.020 (3)	−0.048 (4)	0.025 (3)
C47	0.081 (4)	0.046 (3)	0.077 (4)	0.011 (3)	−0.047 (4)	−0.014 (3)
C48	0.063 (3)	0.098 (5)	0.042 (3)	0.012 (3)	−0.013 (2)	−0.007 (3)
C49	0.051 (3)	0.061 (3)	0.043 (3)	−0.008 (2)	−0.011 (2)	0.015 (2)
N51	0.056 (2)	0.0243 (17)	0.0323 (17)	0.0045 (15)	−0.0109 (15)	−0.0048 (14)
C51	0.063 (3)	0.027 (2)	0.050 (3)	0.002 (2)	−0.025 (2)	−0.0061 (19)
C52	0.050 (2)	0.024 (2)	0.037 (2)	0.0029 (17)	−0.0067 (18)	−0.0066 (16)
O52	0.0610 (19)	0.0221 (15)	0.0485 (17)	−0.0033 (13)	−0.0129 (15)	−0.0027 (12)
C53	0.109 (5)	0.031 (2)	0.057 (3)	0.013 (3)	−0.049 (3)	−0.015 (2)
C54	0.116 (5)	0.038 (3)	0.036 (2)	0.030 (3)	−0.029 (3)	−0.020 (2)
C55	0.073 (3)	0.039 (3)	0.030 (2)	0.014 (2)	−0.006 (2)	−0.0089 (18)
O54	0.167 (5)	0.067 (3)	0.038 (2)	0.039 (3)	−0.034 (3)	−0.0287 (19)
N61	0.050 (2)	0.0212 (16)	0.0314 (17)	−0.0011 (14)	−0.0078 (15)	−0.0018 (13)
C61	0.050 (2)	0.0215 (18)	0.0265 (18)	−0.0017 (16)	−0.0055 (17)	0.0002 (14)
C62	0.045 (2)	0.0219 (19)	0.0307 (19)	−0.0004 (15)	−0.0020 (16)	−0.0014 (15)
O62	0.072 (2)	0.0244 (15)	0.0324 (15)	−0.0064 (14)	0.0010 (14)	−0.0036 (11)
C63	0.051 (2)	0.0250 (19)	0.0292 (19)	−0.0003 (17)	−0.0016 (17)	−0.0025 (15)
C64	0.058 (3)	0.032 (2)	0.032 (2)	−0.0028 (19)	0.0043 (18)	−0.0070 (17)
C65	0.054 (3)	0.037 (2)	0.038 (2)	0.006 (2)	−0.007 (2)	−0.0021 (18)
N71	0.0511 (19)	0.0186 (15)	0.0277 (16)	−0.0014 (13)	−0.0014 (14)	−0.0014 (12)
C71	0.056 (2)	0.0192 (18)	0.0270 (19)	0.0012 (16)	−0.0039 (17)	−0.0025 (14)
C72	0.054 (2)	0.0245 (19)	0.0250 (17)	−0.0018 (17)	−0.0003 (17)	−0.0030 (14)
O72	0.0616 (19)	0.0170 (14)	0.0445 (16)	−0.0011 (12)	−0.0110 (14)	0.0037 (11)
C73	0.051 (2)	0.029 (2)	0.031 (2)	−0.0003 (17)	0.0000 (17)	−0.0017 (16)
C74	0.063 (3)	0.033 (2)	0.029 (2)	0.003 (2)	0.0019 (19)	−0.0007 (16)
C75	0.077 (3)	0.038 (3)	0.037 (2)	0.009 (2)	0.006 (2)	−0.0078 (19)
C76	0.082 (4)	0.056 (3)	0.040 (3)	−0.002 (3)	0.016 (3)	0.002 (2)
N81	0.0492 (19)	0.0203 (16)	0.0354 (17)	0.0005 (14)	−0.0022 (15)	−0.0006 (13)
C81	0.053 (2)	0.0214 (19)	0.041 (2)	0.0009 (17)	−0.0061 (19)	−0.0034 (16)
C82	0.064 (3)	0.025 (2)	0.042 (2)	−0.0007 (19)	−0.010 (2)	−0.0006 (18)
O82	0.126 (4)	0.0357 (19)	0.046 (2)	0.027 (2)	−0.014 (2)	0.0070 (16)
C83	0.051 (2)	0.031 (2)	0.050 (3)	0.0001 (19)	0.000 (2)	−0.0081 (18)
C84	0.069 (3)	0.036 (2)	0.052 (3)	0.005 (2)	0.007 (3)	−0.006 (2)
C85	0.045 (4)	0.046 (4)	0.050 (4)	0.014 (3)	−0.003 (3)	−0.010 (3)
C86	0.062 (6)	0.054 (6)	0.079 (7)	−0.016 (5)	0.033 (5)	−0.019 (5)
C85'	0.084 (11)	0.039 (8)	0.041 (7)	0.006 (7)	0.006 (7)	0.002 (6)
C86'	0.13 (2)	0.069 (14)	0.080 (14)	0.028 (15)	0.025 (14)	0.023 (11)
N91	0.059 (2)	0.0304 (18)	0.0287 (17)	−0.0014 (16)	−0.0072 (16)	−0.0002 (14)
C91	0.053 (2)	0.035 (2)	0.030 (2)	−0.0077 (19)	−0.0081 (18)	0.0026 (16)
C92	0.041 (2)	0.039 (2)	0.0241 (17)	−0.0042 (17)	−0.0020 (15)	0.0027 (16)
O92	0.0416 (17)	0.0433 (18)	0.0484 (18)	−0.0013 (13)	−0.0074 (14)	−0.0005 (14)
C93	0.067 (3)	0.040 (3)	0.039 (2)	−0.015 (2)	−0.011 (2)	0.004 (2)
C94	0.060 (3)	0.032 (2)	0.040 (2)	−0.014 (2)	−0.012 (2)	0.0049 (18)
C95	0.064 (3)	0.038 (2)	0.038 (2)	−0.007 (2)	−0.007 (2)	0.0040 (19)
C96	0.072 (3)	0.044 (3)	0.044 (3)	−0.012 (2)	−0.013 (2)	0.011 (2)
C97	0.073 (3)	0.048 (3)	0.039 (2)	−0.015 (3)	−0.004 (2)	0.009 (2)
C98	0.068 (3)	0.055 (3)	0.050 (3)	−0.014 (3)	0.005 (3)	0.000 (2)
C99	0.061 (3)	0.038 (3)	0.053 (3)	−0.007 (2)	−0.005 (2)	0.008 (2)
N101	0.0384 (17)	0.0260 (17)	0.0270 (15)	0.0007 (13)	−0.0027 (13)	0.0006 (12)
C101	0.043 (2)	0.0246 (19)	0.0265 (18)	0.0016 (15)	0.0006 (15)	0.0010 (14)
C102	0.041 (2)	0.0241 (19)	0.0259 (18)	0.0001 (15)	0.0002 (15)	0.0001 (14)
O102	0.070 (2)	0.0264 (15)	0.0336 (15)	0.0071 (14)	0.0053 (14)	−0.0031 (12)
C103	0.056 (3)	0.036 (2)	0.0258 (19)	−0.0042 (19)	−0.0039 (17)	−0.0008 (16)
C104	0.046 (2)	0.036 (2)	0.0281 (18)	−0.0054 (17)	−0.0072 (17)	0.0022 (16)
C105	0.046 (2)	0.031 (2)	0.036 (2)	0.0007 (17)	−0.0071 (18)	0.0025 (16)
O104	0.0499 (18)	0.050 (2)	0.0432 (17)	−0.0073 (15)	−0.0152 (15)	0.0111 (15)
O1M	0.061 (2)	0.067 (3)	0.0432 (19)	−0.0003 (19)	−0.0035 (16)	0.0120 (17)
C1M	0.077 (4)	0.115 (6)	0.067 (4)	0.032 (4)	0.024 (3)	0.041 (4)
O1W	0.117 (4)	0.042 (2)	0.049 (2)	−0.008 (2)	0.006 (2)	0.0042 (17)
O2M	0.156 (9)	0.38 (2)	0.144 (9)	0.047 (12)	0.009 (7)	0.065 (11)
C2M	0.178 (13)	0.209 (16)	0.152 (12)	0.029 (12)	−0.047 (10)	0.069 (12)

### cyclo(Val-Leu-Leu-D-Phe-tHyp)2 methanol disolvate monohydrate (4) Geometric parameters (Å, º)

**Table:**

N11—C102	1.336 (5)	C63—H63	0.9800
N11—C11	1.458 (5)	C64—H64A	0.9600
N11—H11	0.8600	C64—H64B	0.9600
C11—C12	1.533 (5)	C64—H64C	0.9600
C11—C13	1.546 (6)	C65—H65A	0.9600
C11—H11A	0.9800	C65—H65B	0.9600
C12—O12	1.231 (5)	C65—H65C	0.9600
C12—N21	1.331 (5)	N71—C71	1.471 (5)
C13—C15	1.519 (6)	N71—H71	0.8600
C13—C14	1.519 (7)	C71—C72	1.529 (6)
C13—H13	0.9800	C71—C73	1.528 (6)
C14—H14A	0.9600	C71—H71A	0.9800
C14—H14B	0.9600	C72—O72	1.230 (5)
C14—H14C	0.9600	C72—N81	1.338 (6)
C15—H15A	0.9600	C73—C74	1.532 (6)
C15—H15B	0.9600	C73—H73A	0.9700
C15—H15C	0.9600	C73—H73B	0.9700
N21—C21	1.468 (5)	C74—C76	1.509 (8)
N21—H21	0.8600	C74—C75	1.539 (7)
C21—C22	1.521 (5)	C74—H74	0.9800
C21—C23	1.530 (6)	C75—H75A	0.9600
C21—H21A	0.9800	C75—H75B	0.9600
C22—O22	1.234 (5)	C75—H75C	0.9600
C22—N31	1.350 (5)	C76—H76A	0.9600
C23—C24	1.533 (6)	C76—H76B	0.9600
C23—H23A	0.9700	C76—H76C	0.9600
C23—H23B	0.9700	N81—C81	1.458 (6)
C24—C25	1.474 (9)	N81—H81	0.8600
C24—C26	1.523 (8)	C81—C82	1.532 (7)
C24—H24	0.9800	C81—C83	1.540 (7)
C25—H25A	0.9600	C81—H81A	0.9800
C25—H25B	0.9600	C82—O82	1.225 (6)
C25—H25C	0.9600	C82—N91	1.333 (7)
C26—H26A	0.9600	C83—C84	1.520 (7)
C26—H26B	0.9600	C83—H83A	0.9700
C26—H26C	0.9600	C83—H83B	0.9700
N31—C31	1.462 (5)	C84—C85'	1.402 (12)
N31—H31	0.8600	C84—C86	1.456 (11)
C31—C32	1.534 (5)	C84—C85	1.508 (9)
C31—C33	1.529 (7)	C84—C86'	1.642 (15)
C31—H31A	0.9800	C84—H84	0.9800
C32—O32	1.237 (5)	C85—H85A	0.9600
C32—N41	1.335 (6)	C85—H85B	0.9600
C33—C34	1.536 (6)	C85—H85C	0.9600
C33—H33A	0.9700	C86—H86A	0.9600
C33—H33B	0.9700	C86—H86B	0.9600
C34—C36	1.512 (9)	C86—H86C	0.9600
C34—C35	1.515 (8)	C85'—H85D	0.9600
C34—H34	0.9800	C85'—H85E	0.9600
C35—H35A	0.9600	C85'—H85F	0.9600
C35—H35B	0.9600	C86'—H86D	0.9600
C35—H35C	0.9600	C86'—H86E	0.9600
C36—H36A	0.9600	C86'—H86F	0.9600
C36—H36B	0.9600	N91—C91	1.463 (6)
C36—H36C	0.9600	N91—H91	0.8600
N41—C41	1.460 (5)	C91—C92	1.526 (6)
N41—H41	0.8600	C91—C93	1.533 (7)
C41—C42	1.528 (6)	C91—H91A	0.9800
C41—C43	1.531 (6)	C92—O92	1.223 (6)
C41—H41A	0.9800	C92—N101	1.344 (6)
C42—O42	1.220 (6)	C93—C94	1.510 (7)
C42—N51	1.346 (5)	C93—H93A	0.9700
C43—C44	1.515 (6)	C93—H93B	0.9700
C43—H43A	0.9700	C94—C95	1.406 (8)
C43—H43B	0.9700	C94—C99	1.380 (8)
C44—C45	1.384 (7)	C95—C96	1.394 (7)
C44—C49	1.376 (7)	C95—H95	0.9300
C45—C46	1.391 (9)	C96—C97	1.365 (9)
C45—H45	0.9300	C96—H96	0.9300
C46—C47	1.368 (11)	C97—C98	1.376 (9)
C46—H46	0.9300	C97—H97	0.9300
C47—C48	1.356 (11)	C98—C99	1.397 (8)
C47—H47	0.9300	C98—H98	0.9300
C48—C49	1.392 (8)	C99—H99	0.9300
C48—H48	0.9300	N101—C101	1.465 (5)
C49—H49	0.9300	N101—C105	1.474 (6)
N51—C51	1.459 (6)	C101—C102	1.528 (5)
N51—C55	1.476 (6)	C101—C103	1.533 (6)
C51—C53	1.543 (7)	C101—H101	0.9800
C51—C52	1.544 (6)	C102—O102	1.235 (5)
C51—H51	0.9800	C103—C104	1.530 (7)
C52—O52	1.237 (6)	C103—H10A	0.9700
C52—N61	1.339 (6)	C103—H10B	0.9700
C53—C54	1.521 (11)	C104—O104	1.419 (5)
C53—H53A	0.9700	C104—C105	1.529 (6)
C53—H53B	0.9700	C104—H104	0.9800
C54—O54	1.422 (6)	C105—H10C	0.9700
C54—C55	1.534 (7)	C105—H10D	0.9700
C54—H54A	0.9800	O104—H10E	0.8200
C55—H55A	0.9700	O1M—C1M	1.412 (8)
C55—H55B	0.9700	O1M—H1M	0.8200
O54—H54	0.8200	C1M—H1M1	0.9600
N61—C61	1.460 (5)	C1M—H1M2	0.9600
N61—H61	0.8600	C1M—H1M3	0.9600
C61—C62	1.536 (5)	O1W—H1W	0.89 (2)
C61—C63	1.533 (6)	O1W—H2W	0.86 (3)
C61—H61A	0.9800	O2M—C2M	1.401 (11)
C62—O62	1.229 (5)	O2M—H2MA	0.87 (3)
C62—N71	1.342 (5)	C2M—H2M1	0.9600
C63—C64	1.527 (6)	C2M—H2M2	0.9600
C63—C65	1.532 (6)	C2M—H2M3	0.9600
			
C102—N11—C11	123.9 (3)	C64—C63—H63	108.2
C102—N11—H11	118.1	C65—C63—H63	108.2
C11—N11—H11	118.1	C61—C63—H63	108.2
N11—C11—C12	107.4 (3)	C63—C64—H64A	109.5
N11—C11—C13	110.5 (3)	C63—C64—H64B	109.5
C12—C11—C13	110.7 (3)	H64A—C64—H64B	109.5
N11—C11—H11A	109.4	C63—C64—H64C	109.5
C12—C11—H11A	109.4	H64A—C64—H64C	109.5
C13—C11—H11A	109.4	H64B—C64—H64C	109.5
O12—C12—N21	124.2 (4)	C63—C65—H65A	109.5
O12—C12—C11	120.0 (3)	C63—C65—H65B	109.5
N21—C12—C11	115.7 (3)	H65A—C65—H65B	109.5
C15—C13—C14	109.9 (4)	C63—C65—H65C	109.5
C15—C13—C11	110.9 (4)	H65A—C65—H65C	109.5
C14—C13—C11	111.2 (4)	H65B—C65—H65C	109.5
C15—C13—H13	108.2	C62—N71—C71	121.9 (3)
C14—C13—H13	108.2	C62—N71—H71	119.0
C11—C13—H13	108.2	C71—N71—H71	119.0
C13—C14—H14A	109.5	N71—C71—C72	106.6 (3)
C13—C14—H14B	109.5	N71—C71—C73	110.8 (3)
H14A—C14—H14B	109.5	C72—C71—C73	110.6 (3)
C13—C14—H14C	109.5	N71—C71—H71A	109.6
H14A—C14—H14C	109.5	C72—C71—H71A	109.6
H14B—C14—H14C	109.5	C73—C71—H71A	109.6
C13—C15—H15A	109.5	O72—C72—N81	122.6 (4)
C13—C15—H15B	109.5	O72—C72—C71	121.6 (4)
H15A—C15—H15B	109.5	N81—C72—C71	115.7 (4)
C13—C15—H15C	109.5	C71—C73—C74	114.7 (4)
H15A—C15—H15C	109.5	C71—C73—H73A	108.6
H15B—C15—H15C	109.5	C74—C73—H73A	108.6
C12—N21—C21	123.0 (3)	C71—C73—H73B	108.6
C12—N21—H21	118.5	C74—C73—H73B	108.6
C21—N21—H21	118.5	H73A—C73—H73B	107.6
N21—C21—C22	105.4 (3)	C76—C74—C73	110.5 (4)
N21—C21—C23	108.9 (3)	C76—C74—C75	111.6 (4)
C22—C21—C23	112.9 (3)	C73—C74—C75	112.3 (4)
N21—C21—H21A	109.8	C76—C74—H74	107.4
C22—C21—H21A	109.8	C73—C74—H74	107.4
C23—C21—H21A	109.8	C75—C74—H74	107.4
O22—C22—N31	123.4 (4)	C74—C75—H75A	109.5
O22—C22—C21	119.8 (4)	C74—C75—H75B	109.5
N31—C22—C21	116.7 (3)	H75A—C75—H75B	109.5
C24—C23—C21	117.2 (4)	C74—C75—H75C	109.5
C24—C23—H23A	108.0	H75A—C75—H75C	109.5
C21—C23—H23A	108.0	H75B—C75—H75C	109.5
C24—C23—H23B	108.0	C74—C76—H76A	109.5
C21—C23—H23B	108.0	C74—C76—H76B	109.5
H23A—C23—H23B	107.2	H76A—C76—H76B	109.5
C25—C24—C26	111.5 (5)	C74—C76—H76C	109.5
C25—C24—C23	118.5 (5)	H76A—C76—H76C	109.5
C26—C24—C23	108.4 (4)	H76B—C76—H76C	109.5
C25—C24—H24	105.8	C72—N81—C81	121.3 (4)
C26—C24—H24	105.8	C72—N81—H81	119.4
C23—C24—H24	105.8	C81—N81—H81	119.4
C24—C25—H25A	109.5	N81—C81—C82	111.2 (4)
C24—C25—H25B	109.5	N81—C81—C83	110.7 (4)
H25A—C25—H25B	109.5	C82—C81—C83	109.2 (4)
C24—C25—H25C	109.5	N81—C81—H81A	108.6
H25A—C25—H25C	109.5	C82—C81—H81A	108.6
H25B—C25—H25C	109.5	C83—C81—H81A	108.6
C24—C26—H26A	109.5	O82—C82—N91	122.5 (5)
C24—C26—H26B	109.5	O82—C82—C81	120.6 (5)
H26A—C26—H26B	109.5	N91—C82—C81	116.9 (4)
C24—C26—H26C	109.5	C81—C83—C84	114.8 (4)
H26A—C26—H26C	109.5	C81—C83—H83A	108.6
H26B—C26—H26C	109.5	C84—C83—H83A	108.6
C22—N31—C31	121.9 (3)	C81—C83—H83B	108.6
C22—N31—H31	119.1	C84—C83—H83B	108.6
C31—N31—H31	119.1	H83A—C83—H83B	107.5
N31—C31—C32	108.4 (3)	C86—C84—C85	114.9 (6)
N31—C31—C33	111.1 (3)	C85'—C84—C83	118.7 (7)
C32—C31—C33	109.1 (3)	C86—C84—C83	112.2 (6)
N31—C31—H31A	109.4	C85—C84—C83	111.5 (5)
C32—C31—H31A	109.4	C85'—C84—C86'	105.8 (9)
C33—C31—H31A	109.4	C83—C84—C86'	102.4 (8)
O32—C32—N41	122.0 (4)	C86—C84—H84	105.8
O32—C32—C31	120.6 (4)	C85—C84—H84	105.8
N41—C32—C31	117.4 (3)	C83—C84—H84	105.8
C34—C33—C31	114.9 (4)	C84—C85—H85A	109.5
C34—C33—H33A	108.5	C84—C85—H85B	109.5
C31—C33—H33A	108.5	H85A—C85—H85B	109.5
C34—C33—H33B	108.5	C84—C85—H85C	109.5
C31—C33—H33B	108.5	H85A—C85—H85C	109.5
H33A—C33—H33B	107.5	H85B—C85—H85C	109.5
C36—C34—C35	111.5 (5)	C84—C86—H86A	109.5
C36—C34—C33	110.3 (5)	C84—C86—H86B	109.5
C35—C34—C33	112.2 (4)	H86A—C86—H86B	109.5
C36—C34—H34	107.5	C84—C86—H86C	109.5
C35—C34—H34	107.5	H86A—C86—H86C	109.5
C33—C34—H34	107.5	H86B—C86—H86C	109.5
C34—C35—H35A	109.5	C84—C85'—H85D	109.5
C34—C35—H35B	109.5	C84—C85'—H85E	109.5
H35A—C35—H35B	109.5	H85D—C85'—H85E	109.5
C34—C35—H35C	109.5	C84—C85'—H85F	109.5
H35A—C35—H35C	109.5	H85D—C85'—H85F	109.5
H35B—C35—H35C	109.5	H85E—C85'—H85F	109.5
C34—C36—H36A	109.5	C84—C86'—H86D	109.5
C34—C36—H36B	109.5	C84—C86'—H86E	109.5
H36A—C36—H36B	109.5	H86D—C86'—H86E	109.5
C34—C36—H36C	109.5	C84—C86'—H86F	109.5
H36A—C36—H36C	109.5	H86D—C86'—H86F	109.5
H36B—C36—H36C	109.5	H86E—C86'—H86F	109.5
C32—N41—C41	119.1 (3)	C82—N91—C91	121.3 (4)
C32—N41—H41	120.5	C82—N91—H91	119.3
C41—N41—H41	120.5	C91—N91—H91	119.3
N41—C41—C42	109.8 (3)	N91—C91—C92	105.9 (3)
N41—C41—C43	110.4 (3)	N91—C91—C93	112.5 (4)
C42—C41—C43	109.0 (4)	C92—C91—C93	112.7 (4)
N41—C41—H41A	109.2	N91—C91—H91A	108.5
C42—C41—H41A	109.2	C92—C91—H91A	108.5
C43—C41—H41A	109.2	C93—C91—H91A	108.5
O42—C42—N51	122.2 (4)	O92—C92—N101	123.1 (4)
O42—C42—C41	120.1 (4)	O92—C92—C91	120.7 (4)
N51—C42—C41	117.7 (4)	N101—C92—C91	116.0 (4)
C44—C43—C41	109.6 (4)	C94—C93—C91	110.7 (4)
C44—C43—H43A	109.7	C94—C93—H93A	109.5
C41—C43—H43A	109.7	C91—C93—H93A	109.5
C44—C43—H43B	109.7	C94—C93—H93B	109.5
C41—C43—H43B	109.7	C91—C93—H93B	109.5
H43A—C43—H43B	108.2	H93A—C93—H93B	108.1
C45—C44—C49	118.0 (5)	C95—C94—C99	118.7 (5)
C45—C44—C43	121.6 (5)	C95—C94—C93	119.7 (5)
C49—C44—C43	120.3 (4)	C99—C94—C93	121.7 (5)
C44—C45—C46	120.5 (6)	C96—C95—C94	120.4 (5)
C44—C45—H45	119.8	C96—C95—H95	119.8
C46—C45—H45	119.8	C94—C95—H95	119.8
C47—C46—C45	120.7 (6)	C95—C96—C97	119.7 (6)
C47—C46—H46	119.7	C95—C96—H96	120.1
C45—C46—H46	119.7	C97—C96—H96	120.1
C46—C47—C48	119.1 (5)	C96—C97—C98	120.7 (5)
C46—C47—H47	120.4	C96—C97—H97	119.7
C48—C47—H47	120.4	C98—C97—H97	119.7
C47—C48—C49	120.9 (6)	C99—C98—C97	120.1 (6)
C47—C48—H48	119.6	C99—C98—H98	119.9
C49—C48—H48	119.6	C97—C98—H98	119.9
C44—C49—C48	120.7 (5)	C98—C99—C94	120.3 (5)
C44—C49—H49	119.6	C98—C99—H99	119.8
C48—C49—H49	119.6	C94—C99—H99	119.8
C42—N51—C51	120.7 (4)	C92—N101—C101	119.7 (3)
C42—N51—C55	126.5 (4)	C92—N101—C105	128.1 (4)
C51—N51—C55	112.5 (4)	C101—N101—C105	112.2 (3)
N51—C51—C53	101.6 (4)	N101—C101—C102	113.0 (3)
N51—C51—C52	114.6 (4)	N101—C101—C103	103.4 (3)
C53—C51—C52	111.8 (4)	C102—C101—C103	112.4 (3)
N51—C51—H51	109.5	N101—C101—H101	109.3
C53—C51—H51	109.5	C102—C101—H101	109.3
C52—C51—H51	109.5	C103—C101—H101	109.3
O52—C52—N61	124.4 (4)	O102—C102—N11	123.9 (4)
O52—C52—C51	118.6 (4)	O102—C102—C101	119.1 (3)
N61—C52—C51	116.9 (4)	N11—C102—C101	117.0 (3)
C51—C53—C54	103.4 (4)	C104—C103—C101	104.2 (3)
C51—C53—H53A	111.1	C104—C103—H10A	110.9
C54—C53—H53A	111.1	C101—C103—H10A	110.9
C51—C53—H53B	111.1	C104—C103—H10B	110.9
C54—C53—H53B	111.1	C101—C103—H10B	110.9
H53A—C53—H53B	109.0	H10A—C103—H10B	108.9
O54—C54—C55	113.6 (5)	O104—C104—C103	109.8 (4)
O54—C54—C53	110.8 (6)	O104—C104—C105	112.1 (4)
C55—C54—C53	102.5 (5)	C103—C104—C105	104.1 (4)
O54—C54—H54A	109.9	O104—C104—H104	110.2
C55—C54—H54A	109.9	C103—C104—H104	110.2
C53—C54—H54A	109.9	C105—C104—H104	110.2
N51—C55—C54	103.6 (5)	N101—C105—C104	104.6 (3)
N51—C55—H55A	111.0	N101—C105—H10C	110.8
C54—C55—H55A	111.0	C104—C105—H10C	110.8
N51—C55—H55B	111.0	N101—C105—H10D	110.8
C54—C55—H55B	111.0	C104—C105—H10D	110.8
H55A—C55—H55B	109.0	H10C—C105—H10D	108.9
C54—O54—H54	109.5	C104—O104—H10E	109.5
C52—N61—C61	122.4 (3)	C1M—O1M—H1M	109.5
C52—N61—H61	118.8	O1M—C1M—H1M1	109.5
C61—N61—H61	118.8	O1M—C1M—H1M2	109.5
N61—C61—C62	109.7 (3)	H1M1—C1M—H1M2	109.5
N61—C61—C63	111.0 (3)	O1M—C1M—H1M3	109.5
C62—C61—C63	109.1 (3)	H1M1—C1M—H1M3	109.5
N61—C61—H61A	109.0	H1M2—C1M—H1M3	109.5
C62—C61—H61A	109.0	H1W—O1W—H2W	103 (5)
C63—C61—H61A	109.0	C2M—O2M—H2MA	132 (7)
O62—C62—N71	123.2 (4)	O2M—C2M—H2M1	109.5
O62—C62—C61	121.8 (4)	O2M—C2M—H2M2	109.5
N71—C62—C61	114.9 (3)	H2M1—C2M—H2M2	109.5
C64—C63—C65	111.2 (4)	O2M—C2M—H2M3	109.5
C64—C63—C61	110.1 (4)	H2M1—C2M—H2M3	109.5
C65—C63—C61	110.8 (4)	H2M2—C2M—H2M3	109.5
			
C102—N11—C11—C12	−120.8 (4)	C52—N61—C61—C63	119.9 (4)
C102—N11—C11—C13	118.3 (4)	N61—C61—C62—O62	−62.7 (5)
N11—C11—C12—O12	−41.0 (5)	C63—C61—C62—O62	59.1 (5)
C13—C11—C12—O12	79.7 (4)	N61—C61—C62—N71	120.1 (4)
N11—C11—C12—N21	142.0 (3)	C63—C61—C62—N71	−118.1 (4)
C13—C11—C12—N21	−97.2 (4)	N61—C61—C63—C64	−59.8 (4)
N11—C11—C13—C15	−175.6 (4)	C62—C61—C63—C64	179.2 (3)
C12—C11—C13—C15	65.5 (5)	N61—C61—C63—C65	176.7 (3)
N11—C11—C13—C14	−53.0 (5)	C62—C61—C63—C65	55.7 (4)
C12—C11—C13—C14	−171.9 (4)	O62—C62—N71—C71	−6.6 (7)
O12—C12—N21—C21	1.4 (6)	C61—C62—N71—C71	170.6 (4)
C11—C12—N21—C21	178.2 (3)	C62—N71—C71—C72	−98.0 (4)
C12—N21—C21—C22	−129.5 (4)	C62—N71—C71—C73	141.6 (4)
C12—N21—C21—C23	109.0 (4)	N71—C71—C72—O72	−72.7 (5)
N21—C21—C22—O22	−61.4 (5)	C73—C71—C72—O72	47.7 (5)
C23—C21—C22—O22	57.4 (5)	N71—C71—C72—N81	107.2 (4)
N21—C21—C22—N31	115.4 (4)	C73—C71—C72—N81	−132.4 (4)
C23—C21—C22—N31	−125.8 (4)	N71—C71—C73—C74	174.7 (3)
N21—C21—C23—C24	−174.0 (4)	C72—C71—C73—C74	56.8 (5)
C22—C21—C23—C24	69.2 (5)	C71—C73—C74—C76	−171.1 (4)
C21—C23—C24—C25	−57.2 (7)	C71—C73—C74—C75	63.5 (6)
C21—C23—C24—C26	174.5 (4)	O72—C72—N81—C81	−2.5 (6)
O22—C22—N31—C31	−0.1 (6)	C71—C72—N81—C81	177.6 (4)
C21—C22—N31—C31	−176.7 (3)	C72—N81—C81—C82	−80.4 (5)
C22—N31—C31—C32	−120.3 (4)	C72—N81—C81—C83	158.1 (4)
C22—N31—C31—C33	119.8 (4)	N81—C81—C82—O82	150.9 (5)
N31—C31—C32—O32	−82.4 (5)	C83—C81—C82—O82	−86.7 (6)
C33—C31—C32—O32	38.7 (5)	N81—C81—C82—N91	−32.5 (5)
N31—C31—C32—N41	97.3 (4)	C83—C81—C82—N91	89.9 (5)
C33—C31—C32—N41	−141.5 (4)	N81—C81—C83—C84	−63.2 (5)
N31—C31—C33—C34	−59.7 (5)	C82—C81—C83—C84	174.1 (4)
C32—C31—C33—C34	−179.2 (4)	C81—C83—C84—C85'	91.4 (9)
C31—C33—C34—C36	−179.6 (5)	C81—C83—C84—C86	168.9 (7)
C31—C33—C34—C35	−54.6 (6)	C81—C83—C84—C85	−60.5 (7)
O32—C32—N41—C41	−1.1 (6)	C81—C83—C84—C86'	−152.6 (9)
C31—C32—N41—C41	179.2 (4)	O82—C82—N91—C91	−3.0 (8)
C32—N41—C41—C42	56.3 (5)	C81—C82—N91—C91	−179.5 (4)
C32—N41—C41—C43	176.5 (4)	C82—N91—C91—C92	146.8 (4)
N41—C41—C42—O42	57.0 (5)	C82—N91—C91—C93	−89.7 (6)
C43—C41—C42—O42	−64.0 (5)	N91—C91—C92—O92	82.8 (5)
N41—C41—C42—N51	−124.7 (4)	C93—C91—C92—O92	−40.6 (5)
C43—C41—C42—N51	114.3 (4)	N91—C91—C92—N101	−92.4 (4)
N41—C41—C43—C44	175.5 (4)	C93—C91—C92—N101	144.2 (4)
C42—C41—C43—C44	−63.8 (5)	N91—C91—C93—C94	160.7 (4)
C41—C43—C44—C45	91.0 (6)	C92—C91—C93—C94	−79.6 (5)
C41—C43—C44—C49	−85.5 (6)	C91—C93—C94—C95	−68.2 (6)
C49—C44—C45—C46	1.1 (8)	C91—C93—C94—C99	113.0 (5)
C43—C44—C45—C46	−175.5 (5)	C99—C94—C95—C96	2.0 (7)
C44—C45—C46—C47	−1.2 (10)	C93—C94—C95—C96	−176.9 (5)
C45—C46—C47—C48	0.2 (10)	C94—C95—C96—C97	−0.6 (8)
C46—C47—C48—C49	1.0 (10)	C95—C96—C97—C98	−1.4 (8)
C45—C44—C49—C48	0.0 (8)	C96—C97—C98—C99	2.0 (8)
C43—C44—C49—C48	176.6 (5)	C97—C98—C99—C94	−0.6 (8)
C47—C48—C49—C44	−1.1 (10)	C95—C94—C99—C98	−1.4 (7)
O42—C42—N51—C51	5.0 (6)	C93—C94—C99—C98	177.5 (5)
C41—C42—N51—C51	−173.3 (4)	O92—C92—N101—C101	0.1 (6)
O42—C42—N51—C55	178.7 (4)	C91—C92—N101—C101	175.1 (3)
C41—C42—N51—C55	0.5 (6)	O92—C92—N101—C105	176.5 (4)
C42—N51—C51—C53	156.4 (4)	C91—C92—N101—C105	−8.5 (6)
C55—N51—C51—C53	−18.2 (5)	C92—N101—C101—C102	−77.2 (5)
C42—N51—C51—C52	−82.9 (5)	C105—N101—C101—C102	105.9 (4)
C55—N51—C51—C52	102.6 (5)	C92—N101—C101—C103	161.0 (3)
N51—C51—C52—O52	−172.5 (4)	C105—N101—C101—C103	−16.0 (4)
C53—C51—C52—O52	−57.6 (7)	C11—N11—C102—O102	−2.5 (7)
N51—C51—C52—N61	9.2 (6)	C11—N11—C102—C101	178.4 (3)
C53—C51—C52—N61	124.2 (5)	N101—C101—C102—O102	168.4 (4)
N51—C51—C53—C54	35.8 (5)	C103—C101—C102—O102	−75.0 (5)
C52—C51—C53—C54	−86.8 (5)	N101—C101—C102—N11	−12.4 (5)
C51—C53—C54—O54	−161.8 (4)	C103—C101—C102—N11	104.2 (4)
C51—C53—C54—C55	−40.3 (5)	N101—C101—C103—C104	30.5 (4)
C42—N51—C55—C54	179.3 (4)	C102—C101—C103—C104	−91.7 (4)
C51—N51—C55—C54	−6.4 (5)	C101—C103—C104—O104	−154.1 (3)
O54—C54—C55—N51	148.4 (6)	C101—C103—C104—C105	−34.0 (4)
C53—C54—C55—N51	28.8 (5)	C92—N101—C105—C104	178.3 (4)
O52—C52—N61—C61	3.1 (7)	C101—N101—C105—C104	−5.1 (4)
C51—C52—N61—C61	−178.8 (4)	O104—C104—C105—N101	142.7 (4)
C52—N61—C61—C62	−119.4 (4)	C103—C104—C105—N101	24.1 (4)

### cyclo(Val-Leu-Leu-D-Phe-tHyp)2 methanol disolvate monohydrate (4) Hydrogen-bond geometry (Å, º)

**Table:**

*D*—H···*A*	H···*A*	*D*···*A*	*D*—H···*A*
N31—H31···O62	2.01	2.838 (5)	162
N61—H61···O32	2.22	3.048 (5)	160
N81—H81···O12	2.19	2.905 (4)	140
N21—H21···O52^i^	2.25	3.090 (4)	165
N41—H41···O72^i^	2.13	2.966 (5)	165
N71—H71···O22^ii^	1.97	2.818 (4)	168
O104—H10*E*···O102^iii^	2.06	2.869 (5)	168
O54—H54···O2*M*	1.99	2.725 (9)	149
O1*W*—H2*W*···O54	2.01	2.847 (7)	163
O1*W*—H1*W*···O102^ii^	2.03	2.927 (6)	178
O1*M*—H1*M*···O42	2.03	2.831 (5)	166
O2*M*—H2*MA*···O82^iv^	1.99	2.727 (8)	144

Symmetry codes: (i) −*x*+1, *y*+1/2, −*z*+1/2; (ii) −*x*+1, *y*−1/2, −*z*+1/2; (iii) *x*+1/2, −*y*+3/2, −*z*+1; (iv) −*x*+3/2, −*y*+1, *z*−1/2.

### cyclo(Val-Leu-Leu-D-Phe-cHyp)2 monohydrate (5) Crystal data

**Table:**

C_62_H_94_N_10_O_12_·H_2_O	*D*_x_ = 1.190 Mg m^−^^3^
*M**_r_* = 1189.48	Cu *K*α radiation, λ = 1.54184 Å
Orthorhombic, *P*2_1_2_1_2_1_	Cell parameters from 24836 reflections
*a* = 12.0818 (1) Å	θ = 4.3–60.5°
*b* = 19.0030 (2) Å	µ = 0.68 mm^−^^1^
*c* = 28.9218 (2) Å	*T* = 100 K
*V* = 6640.17 (10) Å^3^	Needle, colourless
*Z* = 4	0.40 × 0.10 × 0.05 mm
*F*(000) = 2568	

### cyclo(Val-Leu-Leu-D-Phe-cHyp)2 monohydrate (5) Data collection

**Table:**

RIGAKU Xtalab P200K diffractometer	9942 independent reflections
Radiation source: Rotating-anode	9728 reflections with *I* > 2σ(*I*)
Detector resolution: 5.8140 pixels mm^-1^	*R*_int_ = 0.023
ω–scan	θ_max_ = 60.7°, θ_min_ = 2.8°
Absorption correction: multi-scan (CrysAlisPro; Rigaku OD, 2015)	*h* = −13→13
*T*_min_ = 0.830, *T*_max_ = 1.000	*k* = −21→15
31704 measured reflections	*l* = −30→32

### cyclo(Val-Leu-Leu-D-Phe-cHyp)2 monohydrate (5) Refinement

**Table:**

Refinement on *F*^2^	Hydrogen site location: mixed
Least-squares matrix: full	H-atom parameters constrained
*R*[*F*^2^ > 2σ(*F*^2^)] = 0.030	*w* = 1/[σ^2^(*F*_o_^2^) + (0.0557*P*)^2^ + 1.1936*P*] where *P* = (*F*_o_^2^ + 2*F*_c_^2^)/3
*wR*(*F*^2^) = 0.082	(Δ/σ)_max_ = 0.001
*S* = 1.04	Δρ_max_ = 0.84 e Å^−^^3^
9942 reflections	Δρ_min_ = −0.21 e Å^−^^3^
768 parameters	Absolute structure: Flack *x* determined using 4245 quotients [(*I*^+^)-(*I*^-^)]/[(*I*^+^)+(*I*^-^)] (Parsons et al., 2013)
3 restraints	Absolute structure parameter: 0.00 (3)

### cyclo(Val-Leu-Leu-D-Phe-cHyp)2 monohydrate (5) Special details

**Table:**

Geometry. All esds (except the esd in the dihedral angle between two l.s. planes) are estimated using the full covariance matrix. The cell esds are taken into account individually in the estimation of esds in distances, angles and torsion angles; correlations between esds in cell parameters are only used when they are defined by crystal symmetry. An approximate (isotropic) treatment of cell esds is used for estimating esds involving l.s. planes.

### cyclo(Val-Leu-Leu-D-Phe-cHyp)2 monohydrate (5) Fractional atomic coordinates and isotropic or equivalent isotropic displacement parameters (Å^2^)

**Table:**

	*x*	*y*	*z*	*U*_iso_*/*U*_eq_	
O1W	1.11656 (19)	0.39526 (14)	0.56999 (8)	0.0637 (7)	
H1	1.065590	0.371799	0.556029	0.095*	
H2	1.163177	0.399193	0.544564	0.095*	
N11	0.49693 (15)	0.46552 (9)	0.84981 (6)	0.0172 (4)	
H11	0.500365	0.420578	0.852989	0.021*	
C11	0.56657 (18)	0.49995 (11)	0.81524 (7)	0.0168 (4)	
H11A	0.542514	0.548992	0.812259	0.020*	
C12	0.55215 (17)	0.46381 (11)	0.76838 (7)	0.0161 (4)	
O12	0.57959 (13)	0.40185 (8)	0.76198 (5)	0.0213 (3)	
C13	0.68963 (19)	0.49944 (12)	0.82868 (7)	0.0214 (5)	
H13	0.712888	0.450504	0.833162	0.026*	
C14	0.7063 (2)	0.53910 (13)	0.87403 (8)	0.0262 (5)	
H14A	0.661653	0.518032	0.897732	0.039*	
H14B	0.782870	0.536817	0.882846	0.039*	
H14C	0.685030	0.587383	0.870050	0.039*	
C15	0.7594 (2)	0.53143 (14)	0.79019 (8)	0.0297 (6)	
H15A	0.747532	0.505670	0.762063	0.045*	
H15B	0.738374	0.579668	0.785716	0.045*	
H15C	0.836214	0.529103	0.798513	0.045*	
N21	0.51141 (15)	0.50507 (9)	0.73457 (6)	0.0169 (4)	
H21	0.486367	0.545979	0.741801	0.020*	
C21	0.50792 (18)	0.48343 (11)	0.68628 (7)	0.0162 (4)	
H21A	0.513364	0.432077	0.684214	0.019*	
C22	0.60621 (18)	0.51718 (11)	0.66167 (7)	0.0159 (4)	
O22	0.61337 (13)	0.58172 (8)	0.65797 (6)	0.0235 (3)	
C23	0.40097 (18)	0.50821 (11)	0.66347 (7)	0.0183 (5)	
H23A	0.392852	0.558294	0.668978	0.022*	
H23B	0.339232	0.484715	0.678350	0.022*	
C24	0.39375 (19)	0.49492 (12)	0.61123 (7)	0.0211 (5)	
H24	0.459697	0.515415	0.596804	0.025*	
C25	0.3918 (2)	0.41647 (13)	0.59970 (8)	0.0260 (5)	
H25A	0.455957	0.394149	0.612664	0.039*	
H25B	0.326221	0.395539	0.612442	0.039*	
H25C	0.391952	0.410429	0.566743	0.039*	
C26	0.2926 (2)	0.53148 (13)	0.59097 (9)	0.0304 (6)	
H26A	0.294915	0.580667	0.598479	0.046*	
H26B	0.292371	0.525852	0.557984	0.046*	
H26C	0.226641	0.510962	0.603683	0.046*	
N31	0.68209 (15)	0.47322 (9)	0.64386 (6)	0.0188 (4)	
H31	0.675201	0.428636	0.648097	0.023*	
C31	0.77551 (18)	0.49975 (12)	0.61758 (8)	0.0209 (5)	
H31A	0.786616	0.548872	0.626786	0.025*	
C32	0.75633 (19)	0.49926 (11)	0.56563 (8)	0.0211 (5)	
O32	0.80457 (15)	0.54166 (9)	0.54075 (6)	0.0320 (4)	
C33	0.8826 (2)	0.45997 (13)	0.62899 (9)	0.0276 (5)	
H33A	0.872183	0.410497	0.621980	0.033*	
H33B	0.941432	0.477426	0.609240	0.033*	
C34	0.9188 (2)	0.46708 (14)	0.67944 (9)	0.0313 (6)	
H34	0.861431	0.445911	0.698934	0.038*	
C35	1.0262 (2)	0.42664 (19)	0.68717 (11)	0.0488 (8)	
H35A	1.016180	0.378471	0.678073	0.073*	
H35B	1.084135	0.447380	0.669002	0.073*	
H35C	1.045871	0.428589	0.719302	0.073*	
C36	0.9314 (2)	0.54399 (16)	0.69351 (10)	0.0416 (7)	
H36A	0.862870	0.568296	0.688334	0.062*	
H36B	0.950523	0.546653	0.725685	0.062*	
H36C	0.988787	0.565443	0.675385	0.062*	
N41	0.68870 (16)	0.44902 (10)	0.54849 (6)	0.0220 (4)	
H41	0.662016	0.417547	0.566717	0.026*	
C41	0.66014 (19)	0.44741 (12)	0.49921 (8)	0.0210 (5)	
H41A	0.718002	0.472428	0.482072	0.025*	
C42	0.66122 (18)	0.37014 (12)	0.48399 (7)	0.0191 (5)	
O42	0.57421 (13)	0.33785 (8)	0.47746 (6)	0.0257 (4)	
C43	0.5491 (2)	0.48405 (13)	0.48979 (8)	0.0258 (5)	
H43A	0.489907	0.450059	0.493519	0.031*	
H43B	0.538336	0.520757	0.512653	0.031*	
C44	0.54101 (19)	0.51617 (12)	0.44214 (8)	0.0223 (5)	
C45	0.6101 (2)	0.57210 (12)	0.42997 (8)	0.0255 (5)	
H45	0.662560	0.588141	0.450992	0.031*	
C46	0.6016 (2)	0.60394 (13)	0.38725 (9)	0.0300 (6)	
H46	0.647813	0.641399	0.379782	0.036*	
C47	0.5248 (2)	0.58031 (14)	0.35561 (9)	0.0338 (6)	
H47	0.519164	0.601836	0.326822	0.041*	
C48	0.4562 (2)	0.52472 (15)	0.36664 (9)	0.0343 (6)	
H48	0.404521	0.508567	0.345281	0.041*	
C49	0.4645 (2)	0.49285 (13)	0.40994 (9)	0.0290 (5)	
H49	0.418128	0.455431	0.417288	0.035*	
N51	0.76144 (15)	0.33929 (9)	0.48141 (6)	0.0169 (4)	
C51	0.77331 (19)	0.26394 (11)	0.47106 (7)	0.0189 (5)	
H51	0.722867	0.250970	0.445933	0.023*	
C52	0.75260 (17)	0.21626 (11)	0.51290 (7)	0.0165 (5)	
O52	0.75486 (13)	0.15226 (8)	0.50807 (5)	0.0225 (3)	
C53	0.89348 (19)	0.25888 (12)	0.45410 (7)	0.0217 (5)	
H53A	0.924203	0.212690	0.460343	0.026*	
H53B	0.898342	0.268284	0.421202	0.026*	
C54	0.95295 (18)	0.31523 (12)	0.48180 (7)	0.0199 (5)	
H54	1.021136	0.330168	0.466289	0.024*	
O54	0.97469 (13)	0.29255 (8)	0.52782 (5)	0.0224 (3)	
H54A	1.005417	0.254084	0.527207	0.034*	
C55	0.86995 (18)	0.37504 (12)	0.48432 (8)	0.0201 (5)	
H55A	0.877089	0.400638	0.513182	0.024*	
H55B	0.879863	0.407506	0.458775	0.024*	
N61	0.73065 (15)	0.24835 (9)	0.55318 (6)	0.0170 (4)	
H61	0.726006	0.293502	0.553610	0.020*	
C61	0.71437 (18)	0.21002 (11)	0.59613 (7)	0.0164 (4)	
H61A	0.676135	0.165747	0.589279	0.020*	
C62	0.64029 (17)	0.25485 (11)	0.62684 (7)	0.0160 (4)	
O62	0.64590 (12)	0.32020 (8)	0.62613 (5)	0.0206 (3)	
C63	0.82721 (19)	0.19290 (12)	0.61947 (8)	0.0218 (5)	
H63	0.873429	0.170281	0.595917	0.026*	
C64	0.8141 (2)	0.14023 (14)	0.65875 (8)	0.0300 (6)	
H64A	0.774565	0.099692	0.647811	0.045*	
H64B	0.885854	0.126138	0.669601	0.045*	
H64C	0.773627	0.161631	0.683611	0.045*	
C65	0.8898 (2)	0.25790 (14)	0.63592 (8)	0.0293 (5)	
H65A	0.897357	0.290512	0.610760	0.044*	
H65B	0.849687	0.279811	0.660681	0.044*	
H65C	0.961914	0.244318	0.646670	0.044*	
N71	0.57177 (15)	0.21863 (9)	0.65399 (6)	0.0163 (4)	
H71	0.566887	0.173950	0.649749	0.020*	
C71	0.50404 (18)	0.25023 (11)	0.69074 (7)	0.0165 (4)	
H71A	0.520956	0.300501	0.693403	0.020*	
C72	0.53582 (17)	0.21271 (11)	0.73508 (7)	0.0155 (4)	
O72	0.52627 (13)	0.14809 (8)	0.73774 (5)	0.0216 (3)	
C73	0.38058 (19)	0.24023 (12)	0.68044 (8)	0.0215 (5)	
H73A	0.362406	0.267173	0.652984	0.026*	
H73B	0.367945	0.191009	0.673304	0.026*	
C74	0.3007 (2)	0.26180 (14)	0.71914 (8)	0.0285 (5)	
H74	0.318378	0.234341	0.746859	0.034*	
C75	0.1829 (2)	0.24395 (16)	0.70409 (13)	0.0475 (8)	
H75A	0.178364	0.194813	0.696622	0.071*	
H75B	0.132461	0.254447	0.728794	0.071*	
H75C	0.163759	0.271375	0.677380	0.071*	
C76	0.3089 (2)	0.33960 (13)	0.73107 (9)	0.0292 (5)	
H76A	0.383192	0.350419	0.740498	0.044*	
H76B	0.290034	0.367204	0.704415	0.044*	
H76C	0.258736	0.350276	0.755829	0.044*	
N81	0.57450 (15)	0.25333 (9)	0.76917 (6)	0.0173 (4)	
H81	0.578985	0.297996	0.764751	0.021*	
C81	0.60941 (19)	0.22454 (12)	0.81365 (7)	0.0185 (5)	
H81A	0.594862	0.173811	0.814238	0.022*	
C82	0.53993 (18)	0.26082 (11)	0.85108 (7)	0.0165 (4)	
O82	0.57012 (13)	0.31670 (8)	0.86852 (5)	0.0197 (3)	
C83	0.7324 (2)	0.23763 (13)	0.82220 (8)	0.0261 (5)	
H83A	0.744789	0.288035	0.823312	0.031*	
H83B	0.751505	0.218678	0.852304	0.031*	
C84	0.8107 (2)	0.20600 (13)	0.78637 (9)	0.0280 (5)	
H84	0.792749	0.226641	0.756232	0.034*	
C85	0.7986 (2)	0.12670 (13)	0.78243 (8)	0.0265 (5)	
H85A	0.723296	0.115225	0.774981	0.040*	
H85B	0.846581	0.109536	0.758498	0.040*	
H85C	0.818275	0.105202	0.811320	0.040*	
C86	0.9297 (2)	0.22571 (17)	0.79820 (13)	0.0511 (8)	
H86A	0.935830	0.275951	0.800550	0.077*	
H86B	0.949910	0.204633	0.827152	0.077*	
H86C	0.978217	0.208967	0.774330	0.077*	
N91	0.44509 (16)	0.22898 (9)	0.86294 (6)	0.0203 (4)	
H91	0.425515	0.190375	0.849749	0.024*	
C91	0.37527 (18)	0.26076 (11)	0.89865 (7)	0.0198 (5)	
H91A	0.415204	0.260104	0.928140	0.024*	
C92	0.35026 (18)	0.33736 (11)	0.88548 (7)	0.0184 (5)	
O92	0.30707 (14)	0.35086 (8)	0.84808 (5)	0.0251 (4)	
C93	0.2664 (2)	0.22003 (12)	0.90416 (8)	0.0248 (5)	
H93A	0.218694	0.229021	0.877849	0.030*	
H93B	0.281414	0.169916	0.905414	0.030*	
C94	0.20953 (18)	0.24314 (12)	0.94815 (8)	0.0216 (5)	
C95	0.2366 (2)	0.21121 (13)	0.98984 (9)	0.0299 (6)	
H95	0.284873	0.173110	0.989887	0.036*	
C96	0.1934 (2)	0.23478 (14)	1.03120 (8)	0.0313 (6)	
H96	0.211903	0.212280	1.058685	0.038*	
C97	0.1226 (2)	0.29173 (13)	1.03172 (9)	0.0284 (5)	
H97	0.094492	0.308560	1.059535	0.034*	
C98	0.0941 (2)	0.32349 (14)	0.99045 (9)	0.0335 (6)	
H98	0.045609	0.361525	0.990541	0.040*	
C99	0.1368 (2)	0.29931 (14)	0.94891 (9)	0.0297 (6)	
H99	0.116401	0.321003	0.921376	0.036*	
N101	0.37693 (15)	0.38768 (9)	0.91646 (6)	0.0175 (4)	
C101	0.34819 (19)	0.46171 (11)	0.90807 (8)	0.0203 (5)	
H101	0.273900	0.463679	0.894531	0.024*	
C102	0.42811 (18)	0.50244 (11)	0.87656 (7)	0.0185 (5)	
O102	0.42011 (14)	0.56717 (8)	0.87509 (5)	0.0271 (4)	
C103	0.3431 (2)	0.49189 (13)	0.95715 (8)	0.0281 (5)	
H10A	0.360341	0.541741	0.957243	0.034*	
H10B	0.270457	0.484943	0.970693	0.034*	
C104	0.4303 (2)	0.45028 (12)	0.98296 (8)	0.0266 (5)	
H104	0.418123	0.451855	1.016445	0.032*	
O104	0.53551 (16)	0.47815 (9)	0.97080 (6)	0.0344 (4)	
H10C	0.581010	0.467318	0.990591	0.052*	
C105	0.4174 (2)	0.37607 (12)	0.96414 (7)	0.0208 (5)	
H10D	0.487700	0.351379	0.964094	0.025*	
H10E	0.364330	0.349383	0.982175	0.025*	

### cyclo(Val-Leu-Leu-D-Phe-cHyp)2 monohydrate (5) Atomic displacement parameters (Å^2^)

**Table:**

	*U*^11^	*U*^22^	*U*^33^	*U*^12^	*U*^13^	*U*^23^
O1W	0.0419 (12)	0.0856 (18)	0.0635 (14)	−0.0218 (12)	0.0141 (11)	−0.0452 (13)
N11	0.0227 (9)	0.0110 (8)	0.0179 (9)	0.0013 (7)	0.0039 (7)	0.0019 (7)
C11	0.0218 (11)	0.0122 (10)	0.0164 (10)	0.0010 (9)	0.0011 (9)	0.0021 (8)
C12	0.0150 (10)	0.0137 (11)	0.0196 (11)	−0.0013 (9)	0.0018 (8)	0.0012 (8)
O12	0.0302 (9)	0.0124 (8)	0.0212 (8)	0.0028 (7)	−0.0015 (7)	−0.0002 (6)
C13	0.0218 (12)	0.0214 (11)	0.0210 (11)	0.0011 (9)	−0.0005 (9)	−0.0011 (9)
C14	0.0276 (12)	0.0269 (13)	0.0240 (12)	0.0016 (10)	−0.0052 (10)	−0.0021 (10)
C15	0.0237 (12)	0.0367 (14)	0.0288 (13)	−0.0072 (11)	0.0023 (10)	−0.0034 (11)
N21	0.0221 (10)	0.0120 (9)	0.0165 (9)	0.0030 (7)	0.0017 (7)	−0.0019 (7)
C21	0.0218 (11)	0.0119 (10)	0.0150 (10)	0.0001 (9)	0.0015 (9)	−0.0005 (8)
C22	0.0201 (11)	0.0134 (11)	0.0143 (10)	0.0007 (9)	−0.0028 (9)	−0.0018 (8)
O22	0.0260 (8)	0.0136 (8)	0.0309 (9)	0.0006 (7)	0.0049 (7)	0.0018 (6)
C23	0.0205 (11)	0.0159 (11)	0.0186 (11)	0.0006 (9)	0.0022 (9)	−0.0010 (9)
C24	0.0234 (12)	0.0218 (12)	0.0182 (11)	−0.0015 (9)	−0.0013 (9)	0.0011 (9)
C25	0.0322 (13)	0.0260 (13)	0.0198 (12)	0.0027 (10)	−0.0041 (10)	−0.0041 (9)
C26	0.0378 (14)	0.0267 (13)	0.0267 (13)	0.0023 (11)	−0.0083 (11)	0.0030 (10)
N31	0.0208 (9)	0.0126 (9)	0.0231 (9)	0.0008 (8)	0.0025 (8)	−0.0006 (7)
C31	0.0200 (11)	0.0160 (11)	0.0267 (12)	−0.0014 (9)	0.0054 (9)	−0.0029 (9)
C32	0.0188 (11)	0.0161 (11)	0.0284 (12)	0.0019 (9)	0.0068 (9)	−0.0008 (10)
O32	0.0390 (10)	0.0260 (9)	0.0309 (9)	−0.0088 (8)	0.0072 (8)	0.0024 (7)
C33	0.0209 (11)	0.0290 (13)	0.0330 (13)	0.0010 (10)	0.0026 (10)	−0.0037 (10)
C34	0.0223 (12)	0.0407 (15)	0.0310 (13)	−0.0033 (11)	0.0022 (10)	−0.0023 (11)
C35	0.0313 (15)	0.072 (2)	0.0430 (17)	0.0083 (15)	−0.0082 (13)	−0.0014 (15)
C36	0.0399 (15)	0.0505 (17)	0.0344 (14)	−0.0174 (14)	−0.0004 (12)	−0.0072 (13)
N41	0.0262 (10)	0.0186 (9)	0.0213 (10)	−0.0028 (8)	0.0060 (8)	0.0025 (7)
C41	0.0243 (11)	0.0197 (11)	0.0191 (11)	0.0020 (9)	0.0050 (9)	0.0021 (9)
C42	0.0205 (12)	0.0212 (11)	0.0157 (10)	−0.0011 (10)	0.0010 (9)	0.0053 (9)
O42	0.0203 (8)	0.0251 (8)	0.0316 (9)	−0.0022 (7)	−0.0004 (7)	0.0038 (7)
C43	0.0249 (12)	0.0235 (12)	0.0288 (13)	0.0052 (10)	0.0060 (10)	0.0032 (10)
C44	0.0221 (12)	0.0187 (11)	0.0262 (12)	0.0064 (9)	0.0064 (9)	0.0009 (9)
C45	0.0290 (12)	0.0210 (12)	0.0266 (12)	−0.0017 (10)	0.0017 (10)	−0.0040 (10)
C46	0.0356 (14)	0.0241 (13)	0.0304 (13)	−0.0028 (11)	0.0067 (11)	0.0035 (10)
C47	0.0426 (15)	0.0325 (14)	0.0263 (13)	0.0053 (12)	0.0010 (11)	0.0061 (11)
C48	0.0306 (13)	0.0394 (15)	0.0329 (14)	0.0001 (11)	−0.0047 (11)	−0.0003 (11)
C49	0.0232 (12)	0.0281 (13)	0.0358 (13)	−0.0011 (10)	0.0023 (11)	0.0012 (10)
N51	0.0194 (9)	0.0131 (9)	0.0182 (9)	−0.0016 (7)	0.0008 (7)	0.0008 (7)
C51	0.0226 (11)	0.0177 (11)	0.0164 (10)	−0.0018 (9)	0.0004 (9)	−0.0007 (9)
C52	0.0128 (10)	0.0189 (12)	0.0177 (11)	−0.0029 (8)	−0.0006 (8)	−0.0002 (9)
O52	0.0316 (9)	0.0147 (8)	0.0214 (8)	−0.0001 (6)	0.0022 (7)	−0.0008 (6)
C53	0.0266 (12)	0.0216 (12)	0.0169 (11)	−0.0003 (10)	0.0056 (9)	−0.0015 (9)
C54	0.0184 (11)	0.0223 (12)	0.0189 (11)	−0.0007 (9)	0.0054 (9)	0.0010 (9)
O54	0.0212 (8)	0.0214 (8)	0.0245 (8)	0.0022 (6)	−0.0002 (6)	0.0025 (6)
C55	0.0191 (11)	0.0178 (11)	0.0233 (11)	−0.0040 (9)	0.0028 (9)	0.0020 (9)
N61	0.0211 (9)	0.0129 (9)	0.0169 (9)	−0.0013 (7)	0.0021 (7)	0.0018 (7)
C61	0.0190 (10)	0.0154 (10)	0.0148 (10)	−0.0008 (9)	0.0012 (9)	0.0010 (8)
C62	0.0166 (10)	0.0175 (12)	0.0141 (10)	0.0002 (8)	−0.0029 (8)	0.0009 (8)
O62	0.0255 (8)	0.0126 (8)	0.0237 (8)	−0.0017 (6)	0.0032 (6)	0.0007 (6)
C63	0.0202 (11)	0.0249 (12)	0.0202 (11)	0.0036 (10)	0.0006 (9)	0.0003 (9)
C64	0.0280 (13)	0.0352 (14)	0.0269 (12)	0.0097 (11)	−0.0028 (11)	0.0072 (11)
C65	0.0240 (12)	0.0366 (14)	0.0272 (12)	−0.0012 (11)	−0.0067 (10)	−0.0032 (11)
N71	0.0213 (9)	0.0110 (8)	0.0168 (8)	−0.0003 (7)	0.0037 (7)	−0.0017 (7)
C71	0.0189 (11)	0.0142 (10)	0.0165 (10)	−0.0002 (9)	0.0025 (8)	−0.0017 (8)
C72	0.0160 (10)	0.0138 (11)	0.0167 (10)	0.0004 (8)	0.0050 (8)	−0.0012 (8)
O72	0.0310 (9)	0.0130 (8)	0.0208 (8)	−0.0027 (6)	−0.0023 (7)	0.0005 (6)
C73	0.0217 (11)	0.0185 (11)	0.0245 (12)	0.0013 (9)	−0.0015 (9)	−0.0007 (9)
C74	0.0217 (12)	0.0331 (14)	0.0308 (13)	0.0052 (11)	0.0073 (10)	0.0108 (11)
C75	0.0218 (14)	0.0382 (16)	0.082 (2)	−0.0025 (12)	0.0059 (14)	0.0021 (15)
C76	0.0244 (12)	0.0361 (14)	0.0271 (12)	0.0054 (11)	0.0045 (10)	−0.0015 (11)
N81	0.0259 (10)	0.0102 (9)	0.0159 (9)	0.0014 (8)	0.0009 (8)	−0.0003 (7)
C81	0.0263 (12)	0.0158 (11)	0.0135 (10)	0.0024 (9)	−0.0004 (9)	0.0001 (8)
C82	0.0212 (11)	0.0147 (11)	0.0137 (10)	0.0032 (9)	−0.0025 (8)	0.0022 (8)
O82	0.0250 (8)	0.0149 (8)	0.0193 (7)	0.0000 (6)	0.0007 (6)	−0.0034 (6)
C83	0.0267 (12)	0.0257 (13)	0.0259 (12)	0.0023 (10)	−0.0007 (10)	−0.0051 (10)
C84	0.0275 (13)	0.0255 (13)	0.0310 (13)	0.0028 (10)	0.0041 (11)	−0.0003 (10)
C85	0.0244 (12)	0.0253 (12)	0.0299 (12)	0.0062 (10)	0.0044 (10)	0.0014 (10)
C86	0.0300 (15)	0.0425 (17)	0.081 (2)	−0.0067 (13)	0.0139 (16)	−0.0185 (16)
N91	0.0264 (10)	0.0147 (9)	0.0197 (9)	−0.0018 (8)	0.0052 (8)	−0.0056 (7)
C91	0.0253 (12)	0.0166 (11)	0.0175 (11)	−0.0018 (9)	0.0056 (9)	−0.0020 (9)
C92	0.0204 (11)	0.0184 (11)	0.0165 (11)	−0.0029 (9)	0.0038 (9)	0.0004 (9)
O92	0.0299 (9)	0.0265 (9)	0.0187 (8)	−0.0011 (7)	−0.0017 (7)	0.0000 (6)
C93	0.0276 (12)	0.0211 (12)	0.0257 (12)	−0.0065 (10)	0.0065 (10)	−0.0029 (9)
C94	0.0219 (11)	0.0194 (12)	0.0235 (11)	−0.0063 (9)	0.0032 (9)	−0.0008 (9)
C95	0.0351 (14)	0.0241 (13)	0.0306 (13)	0.0070 (11)	0.0090 (11)	0.0060 (10)
C96	0.0342 (14)	0.0362 (14)	0.0234 (12)	0.0050 (11)	0.0036 (11)	0.0062 (11)
C97	0.0290 (13)	0.0297 (13)	0.0265 (13)	−0.0020 (11)	0.0093 (10)	−0.0046 (10)
C98	0.0293 (13)	0.0336 (14)	0.0376 (14)	0.0089 (11)	0.0070 (11)	0.0020 (11)
C99	0.0269 (13)	0.0351 (14)	0.0270 (13)	0.0032 (11)	0.0011 (10)	0.0073 (11)
N101	0.0234 (9)	0.0127 (9)	0.0165 (9)	0.0008 (8)	0.0025 (7)	0.0006 (7)
C101	0.0246 (11)	0.0143 (11)	0.0221 (11)	0.0028 (9)	0.0059 (9)	0.0016 (9)
C102	0.0228 (11)	0.0152 (12)	0.0177 (10)	0.0019 (9)	−0.0001 (9)	0.0011 (9)
O102	0.0368 (9)	0.0148 (8)	0.0296 (9)	0.0055 (7)	0.0109 (7)	0.0026 (7)
C103	0.0411 (14)	0.0197 (12)	0.0235 (12)	0.0032 (10)	0.0129 (11)	−0.0006 (9)
C104	0.0424 (14)	0.0200 (12)	0.0174 (11)	−0.0076 (11)	0.0039 (10)	−0.0022 (9)
O104	0.0434 (11)	0.0309 (10)	0.0290 (9)	−0.0158 (8)	−0.0058 (8)	0.0015 (7)
C105	0.0276 (12)	0.0184 (11)	0.0165 (10)	−0.0027 (9)	−0.0002 (9)	0.0005 (9)

### cyclo(Val-Leu-Leu-D-Phe-cHyp)2 monohydrate (5) Geometric parameters (Å, º)

**Table:**

O1W—H1	0.861 (2)	C55—H55A	0.9700
O1W—H2	0.929 (2)	C55—H55B	0.9700
N11—C102	1.335 (3)	N61—C61	1.453 (3)
N11—C11	1.461 (3)	N61—H61	0.8600
N11—H11	0.8600	C61—C62	1.522 (3)
C11—C12	1.529 (3)	C61—C63	1.556 (3)
C11—C13	1.537 (3)	C61—H61A	0.9800
C11—H11A	0.9800	C62—O62	1.244 (3)
C12—O12	1.237 (3)	C62—N71	1.333 (3)
C12—N21	1.347 (3)	C63—C64	1.523 (3)
C13—C14	1.526 (3)	C63—C65	1.525 (3)
C13—C15	1.523 (3)	C63—H63	0.9800
C13—H13	0.9800	C64—H64A	0.9600
C14—H14A	0.9600	C64—H64B	0.9600
C14—H14B	0.9600	C64—H64C	0.9600
C14—H14C	0.9600	C65—H65A	0.9600
C15—H15A	0.9600	C65—H65B	0.9600
C15—H15B	0.9600	C65—H65C	0.9600
C15—H15C	0.9600	N71—C71	1.470 (3)
N21—C21	1.456 (3)	N71—H71	0.8600
N21—H21	0.8600	C71—C72	1.517 (3)
C21—C22	1.526 (3)	C71—C73	1.533 (3)
C21—C23	1.525 (3)	C71—H71A	0.9800
C21—H21A	0.9800	C72—O72	1.236 (3)
C22—O22	1.234 (3)	C72—N81	1.337 (3)
C22—N31	1.343 (3)	C73—C74	1.534 (3)
C23—C24	1.534 (3)	C73—H73A	0.9700
C23—H23A	0.9700	C73—H73B	0.9700
C23—H23B	0.9700	C74—C76	1.521 (4)
C24—C25	1.528 (3)	C74—C75	1.527 (4)
C24—C26	1.523 (3)	C74—H74	0.9800
C24—H24	0.9800	C75—H75A	0.9600
C25—H25A	0.9600	C75—H75B	0.9600
C25—H25B	0.9600	C75—H75C	0.9600
C25—H25C	0.9600	C76—H76A	0.9600
C26—H26A	0.9600	C76—H76B	0.9600
C26—H26B	0.9600	C76—H76C	0.9600
C26—H26C	0.9600	N81—C81	1.460 (3)
N31—C31	1.451 (3)	N81—H81	0.8600
N31—H31	0.8600	C81—C83	1.527 (3)
C31—C32	1.520 (3)	C81—C82	1.534 (3)
C31—C33	1.535 (3)	C81—H81A	0.9800
C31—H31A	0.9800	C82—O82	1.231 (3)
C32—O32	1.227 (3)	C82—N91	1.341 (3)
C32—N41	1.351 (3)	C83—C84	1.526 (3)
C33—C34	1.529 (4)	C83—H83A	0.9700
C33—H33A	0.9700	C83—H83B	0.9700
C33—H33B	0.9700	C84—C85	1.518 (3)
C34—C36	1.525 (4)	C84—C86	1.525 (4)
C34—C35	1.524 (4)	C84—H84	0.9800
C34—H34	0.9800	C85—H85A	0.9600
C35—H35A	0.9600	C85—H85B	0.9600
C35—H35B	0.9600	C85—H85C	0.9600
C35—H35C	0.9600	C86—H86A	0.9600
C36—H36A	0.9600	C86—H86B	0.9600
C36—H36B	0.9600	C86—H86C	0.9600
C36—H36C	0.9600	N91—C91	1.464 (3)
N41—C41	1.467 (3)	N91—H91	0.8600
N41—H41	0.8600	C91—C93	1.534 (3)
C41—C42	1.533 (3)	C91—C92	1.535 (3)
C41—C43	1.535 (3)	C91—H91A	0.9800
C41—H41A	0.9800	C92—O92	1.228 (3)
C42—O42	1.232 (3)	C92—N101	1.350 (3)
C42—N51	1.347 (3)	C93—C94	1.511 (3)
C43—C44	1.510 (3)	C93—H93A	0.9700
C43—H43A	0.9700	C93—H93B	0.9700
C43—H43B	0.9700	C94—C99	1.383 (4)
C44—C49	1.385 (4)	C94—C95	1.389 (3)
C44—C45	1.396 (3)	C95—C96	1.380 (4)
C45—C46	1.380 (4)	C95—H95	0.9300
C45—H45	0.9300	C96—C97	1.379 (4)
C46—C47	1.379 (4)	C96—H96	0.9300
C46—H46	0.9300	C97—C98	1.381 (4)
C47—C48	1.380 (4)	C97—H97	0.9300
C47—H47	0.9300	C98—C99	1.386 (4)
C48—C49	1.395 (4)	C98—H98	0.9300
C48—H48	0.9300	C99—H99	0.9300
C49—H49	0.9300	N101—C101	1.469 (3)
N51—C51	1.470 (3)	N101—C105	1.480 (3)
N51—C55	1.479 (3)	C101—C103	1.532 (3)
C51—C52	1.532 (3)	C101—C102	1.537 (3)
C51—C53	1.536 (3)	C101—H101	0.9800
C51—H51	0.9800	C102—O102	1.235 (3)
C52—O52	1.225 (3)	C103—C104	1.514 (4)
C52—N61	1.342 (3)	C103—H10A	0.9700
C53—C54	1.518 (3)	C103—H10B	0.9700
C53—H53A	0.9700	C104—O104	1.422 (3)
C53—H53B	0.9700	C104—C105	1.519 (3)
C54—O54	1.423 (3)	C104—H104	0.9800
C54—C55	1.517 (3)	O104—H10C	0.8200
C54—H54	0.9800	C105—H10D	0.9700
O54—H54A	0.8200	C105—H10E	0.9700
			
H1—O1W—H2	96.0 (2)	C52—N61—C61	122.77 (17)
C102—N11—C11	121.32 (17)	C52—N61—H61	118.6
C102—N11—H11	119.3	C61—N61—H61	118.6
C11—N11—H11	119.3	N61—C61—C62	107.33 (17)
N11—C11—C12	109.86 (17)	N61—C61—C63	110.92 (17)
N11—C11—C13	112.41 (17)	C62—C61—C63	112.29 (17)
C12—C11—C13	109.36 (17)	N61—C61—H61A	108.7
N11—C11—H11A	108.4	C62—C61—H61A	108.7
C12—C11—H11A	108.4	C63—C61—H61A	108.7
C13—C11—H11A	108.4	O62—C62—N71	124.00 (19)
O12—C12—N21	122.92 (19)	O62—C62—C61	121.14 (18)
O12—C12—C11	121.97 (19)	N71—C62—C61	114.86 (18)
N21—C12—C11	115.07 (17)	C64—C63—C65	110.6 (2)
C14—C13—C15	111.0 (2)	C64—C63—C61	111.72 (19)
C14—C13—C11	110.01 (18)	C65—C63—C61	113.64 (19)
C15—C13—C11	110.36 (18)	C64—C63—H63	106.8
C14—C13—H13	108.5	C65—C63—H63	106.8
C15—C13—H13	108.5	C61—C63—H63	106.8
C11—C13—H13	108.5	C63—C64—H64A	109.5
C13—C14—H14A	109.5	C63—C64—H64B	109.5
C13—C14—H14B	109.5	H64A—C64—H64B	109.5
H14A—C14—H14B	109.5	C63—C64—H64C	109.5
C13—C14—H14C	109.5	H64A—C64—H64C	109.5
H14A—C14—H14C	109.5	H64B—C64—H64C	109.5
H14B—C14—H14C	109.5	C63—C65—H65A	109.5
C13—C15—H15A	109.5	C63—C65—H65B	109.5
C13—C15—H15B	109.5	H65A—C65—H65B	109.5
H15A—C15—H15B	109.5	C63—C65—H65C	109.5
C13—C15—H15C	109.5	H65A—C65—H65C	109.5
H15A—C15—H15C	109.5	H65B—C65—H65C	109.5
H15B—C15—H15C	109.5	C62—N71—C71	124.14 (17)
C12—N21—C21	122.86 (17)	C62—N71—H71	117.9
C12—N21—H21	118.6	C71—N71—H71	117.9
C21—N21—H21	118.6	N71—C71—C72	106.17 (16)
N21—C21—C22	107.83 (17)	N71—C71—C73	110.53 (17)
N21—C21—C23	110.62 (17)	C72—C71—C73	110.64 (18)
C22—C21—C23	109.13 (17)	N71—C71—H71A	109.8
N21—C21—H21A	109.7	C72—C71—H71A	109.8
C22—C21—H21A	109.7	C73—C71—H71A	109.8
C23—C21—H21A	109.7	O72—C72—N81	124.11 (19)
O22—C22—N31	122.5 (2)	O72—C72—C71	119.74 (18)
O22—C22—C21	120.84 (19)	N81—C72—C71	116.15 (18)
N31—C22—C21	116.66 (18)	C74—C73—C71	115.93 (19)
C21—C23—C24	115.04 (18)	C74—C73—H73A	108.3
C21—C23—H23A	108.5	C71—C73—H73A	108.3
C24—C23—H23A	108.5	C74—C73—H73B	108.3
C21—C23—H23B	108.5	C71—C73—H73B	108.3
C24—C23—H23B	108.5	H73A—C73—H73B	107.4
H23A—C23—H23B	107.5	C76—C74—C75	110.0 (2)
C25—C24—C26	110.40 (19)	C76—C74—C73	112.6 (2)
C25—C24—C23	112.11 (18)	C75—C74—C73	108.6 (2)
C26—C24—C23	110.44 (19)	C76—C74—H74	108.5
C25—C24—H24	107.9	C75—C74—H74	108.5
C26—C24—H24	107.9	C73—C74—H74	108.5
C23—C24—H24	107.9	C74—C75—H75A	109.5
C24—C25—H25A	109.5	C74—C75—H75B	109.5
C24—C25—H25B	109.5	H75A—C75—H75B	109.5
H25A—C25—H25B	109.5	C74—C75—H75C	109.5
C24—C25—H25C	109.5	H75A—C75—H75C	109.5
H25A—C25—H25C	109.5	H75B—C75—H75C	109.5
H25B—C25—H25C	109.5	C74—C76—H76A	109.5
C24—C26—H26A	109.5	C74—C76—H76B	109.5
C24—C26—H26B	109.5	H76A—C76—H76B	109.5
H26A—C26—H26B	109.5	C74—C76—H76C	109.5
C24—C26—H26C	109.5	H76A—C76—H76C	109.5
H26A—C26—H26C	109.5	H76B—C76—H76C	109.5
H26B—C26—H26C	109.5	C72—N81—C81	122.30 (17)
C22—N31—C31	121.04 (18)	C72—N81—H81	118.8
C22—N31—H31	119.5	C81—N81—H81	118.8
C31—N31—H31	119.5	N81—C81—C83	111.26 (19)
N31—C31—C32	113.39 (19)	N81—C81—C82	107.17 (17)
N31—C31—C33	111.85 (18)	C83—C81—C82	110.20 (18)
C32—C31—C33	109.76 (18)	N81—C81—H81A	109.4
N31—C31—H31A	107.2	C83—C81—H81A	109.4
C32—C31—H31A	107.2	C82—C81—H81A	109.4
C33—C31—H31A	107.2	O82—C82—N91	122.54 (19)
O32—C32—N41	122.4 (2)	O82—C82—C81	121.00 (19)
O32—C32—C31	120.2 (2)	N91—C82—C81	116.46 (18)
N41—C32—C31	117.34 (19)	C84—C83—C81	115.4 (2)
C34—C33—C31	113.7 (2)	C84—C83—H83A	108.4
C34—C33—H33A	108.8	C81—C83—H83A	108.4
C31—C33—H33A	108.8	C84—C83—H83B	108.4
C34—C33—H33B	108.8	C81—C83—H83B	108.4
C31—C33—H33B	108.8	H83A—C83—H83B	107.5
H33A—C33—H33B	107.7	C85—C84—C86	110.6 (2)
C36—C34—C35	111.0 (2)	C85—C84—C83	112.5 (2)
C36—C34—C33	111.6 (2)	C86—C84—C83	109.6 (2)
C35—C34—C33	109.8 (2)	C85—C84—H84	108.0
C36—C34—H34	108.1	C86—C84—H84	108.0
C35—C34—H34	108.1	C83—C84—H84	108.0
C33—C34—H34	108.1	C84—C85—H85A	109.5
C34—C35—H35A	109.5	C84—C85—H85B	109.5
C34—C35—H35B	109.5	H85A—C85—H85B	109.5
H35A—C35—H35B	109.5	C84—C85—H85C	109.5
C34—C35—H35C	109.5	H85A—C85—H85C	109.5
H35A—C35—H35C	109.5	H85B—C85—H85C	109.5
H35B—C35—H35C	109.5	C84—C86—H86A	109.5
C34—C36—H36A	109.5	C84—C86—H86B	109.5
C34—C36—H36B	109.5	H86A—C86—H86B	109.5
H36A—C36—H36B	109.5	C84—C86—H86C	109.5
C34—C36—H36C	109.5	H86A—C86—H86C	109.5
H36A—C36—H36C	109.5	H86B—C86—H86C	109.5
H36B—C36—H36C	109.5	C82—N91—C91	119.13 (18)
C32—N41—C41	120.91 (19)	C82—N91—H91	120.4
C32—N41—H41	119.5	C91—N91—H91	120.4
C41—N41—H41	119.5	N91—C91—C93	111.04 (18)
N41—C41—C42	107.27 (17)	N91—C91—C92	109.25 (17)
N41—C41—C43	111.62 (18)	C93—C91—C92	109.61 (19)
C42—C41—C43	113.01 (19)	N91—C91—H91A	109.0
N41—C41—H41A	108.3	C93—C91—H91A	109.0
C42—C41—H41A	108.3	C92—C91—H91A	109.0
C43—C41—H41A	108.3	O92—C92—N101	122.6 (2)
O42—C42—N51	122.8 (2)	O92—C92—C91	120.03 (19)
O42—C42—C41	120.9 (2)	N101—C92—C91	117.40 (18)
N51—C42—C41	116.14 (19)	C94—C93—C91	109.31 (18)
C44—C43—C41	113.70 (19)	C94—C93—H93A	109.8
C44—C43—H43A	108.8	C91—C93—H93A	109.8
C41—C43—H43A	108.8	C94—C93—H93B	109.8
C44—C43—H43B	108.8	C91—C93—H93B	109.8
C41—C43—H43B	108.8	H93A—C93—H93B	108.3
H43A—C43—H43B	107.7	C99—C94—C95	118.2 (2)
C49—C44—C45	118.2 (2)	C99—C94—C93	121.8 (2)
C49—C44—C43	121.8 (2)	C95—C94—C93	119.8 (2)
C45—C44—C43	119.9 (2)	C96—C95—C94	121.5 (2)
C46—C45—C44	121.0 (2)	C96—C95—H95	119.3
C46—C45—H45	119.5	C94—C95—H95	119.3
C44—C45—H45	119.5	C97—C96—C95	119.9 (2)
C47—C46—C45	120.1 (2)	C97—C96—H96	120.0
C47—C46—H46	120.0	C95—C96—H96	120.0
C45—C46—H46	120.0	C96—C97—C98	119.2 (2)
C46—C47—C48	120.0 (2)	C96—C97—H97	120.4
C46—C47—H47	120.0	C98—C97—H97	120.4
C48—C47—H47	120.0	C99—C98—C97	120.7 (2)
C47—C48—C49	119.8 (2)	C99—C98—H98	119.6
C47—C48—H48	120.1	C97—C98—H98	119.6
C49—C48—H48	120.1	C98—C99—C94	120.4 (2)
C44—C49—C48	120.9 (2)	C98—C99—H99	119.8
C44—C49—H49	119.6	C94—C99—H99	119.8
C48—C49—H49	119.6	C92—N101—C101	120.82 (18)
C42—N51—C51	121.53 (18)	C92—N101—C105	126.31 (18)
C42—N51—C55	126.41 (17)	C101—N101—C105	112.01 (17)
C51—N51—C55	111.87 (17)	N101—C101—C103	102.41 (17)
N51—C51—C52	113.54 (17)	N101—C101—C102	115.58 (18)
N51—C51—C53	102.61 (17)	C103—C101—C102	112.72 (19)
C52—C51—C53	111.69 (18)	N101—C101—H101	108.6
N51—C51—H51	109.6	C103—C101—H101	108.6
C52—C51—H51	109.6	C102—C101—H101	108.6
C53—C51—H51	109.6	O102—C102—N11	123.5 (2)
O52—C52—N61	123.70 (19)	O102—C102—C101	118.23 (19)
O52—C52—C51	119.59 (19)	N11—C102—C101	118.06 (18)
N61—C52—C51	116.70 (18)	C104—C103—C101	103.49 (18)
C54—C53—C51	103.58 (17)	C104—C103—H10A	111.1
C54—C53—H53A	111.0	C101—C103—H10A	111.1
C51—C53—H53A	111.0	C104—C103—H10B	111.1
C54—C53—H53B	111.0	C101—C103—H10B	111.1
C51—C53—H53B	111.0	H10A—C103—H10B	109.0
H53A—C53—H53B	109.0	O104—C104—C103	107.80 (19)
O54—C54—C53	111.53 (18)	O104—C104—C105	110.4 (2)
O54—C54—C55	107.69 (17)	C103—C104—C105	103.72 (19)
C53—C54—C55	103.94 (18)	O104—C104—H104	111.5
O54—C54—H54	111.1	C103—C104—H104	111.5
C53—C54—H54	111.1	C105—C104—H104	111.5
C55—C54—H54	111.1	C104—O104—H10C	109.5
C54—O54—H54A	109.5	N101—C105—C104	103.25 (17)
N51—C55—C54	103.83 (17)	N101—C105—H10D	111.1
N51—C55—H55A	111.0	C104—C105—H10D	111.1
C54—C55—H55A	111.0	N101—C105—H10E	111.1
N51—C55—H55B	111.0	C104—C105—H10E	111.1
C54—C55—H55B	111.0	H10D—C105—H10E	109.1
H55A—C55—H55B	109.0		
			
C102—N11—C11—C12	−128.7 (2)	C52—N61—C61—C62	−153.20 (19)
C102—N11—C11—C13	109.3 (2)	C52—N61—C61—C63	83.8 (2)
N11—C11—C12—O12	−64.0 (3)	N61—C61—C62—O62	−34.2 (3)
C13—C11—C12—O12	59.8 (3)	C63—C61—C62—O62	88.0 (2)
N11—C11—C12—N21	118.24 (19)	N61—C61—C62—N71	146.40 (17)
C13—C11—C12—N21	−117.9 (2)	C63—C61—C62—N71	−91.5 (2)
N11—C11—C13—C14	−61.0 (2)	N61—C61—C63—C64	−168.15 (18)
C12—C11—C13—C14	176.71 (17)	C62—C61—C63—C64	71.8 (2)
N11—C11—C13—C15	176.21 (18)	N61—C61—C63—C65	65.9 (2)
C12—C11—C13—C15	53.9 (2)	C62—C61—C63—C65	−54.2 (2)
O12—C12—N21—C21	−7.4 (3)	O62—C62—N71—C71	−8.1 (3)
C11—C12—N21—C21	170.28 (19)	C61—C62—N71—C71	171.34 (18)
C12—N21—C21—C22	−99.6 (2)	C62—N71—C71—C72	−122.5 (2)
C12—N21—C21—C23	141.16 (19)	C62—N71—C71—C73	117.4 (2)
N21—C21—C22—O22	−64.1 (2)	N71—C71—C72—O72	−57.6 (2)
C23—C21—C22—O22	56.1 (2)	C73—C71—C72—O72	62.4 (2)
N21—C21—C22—N31	117.00 (19)	N71—C71—C72—N81	121.62 (19)
C23—C21—C22—N31	−122.8 (2)	C73—C71—C72—N81	−118.4 (2)
N21—C21—C23—C24	173.98 (17)	N71—C71—C73—C74	172.01 (18)
C22—C21—C23—C24	55.5 (2)	C72—C71—C73—C74	54.7 (3)
C21—C23—C24—C25	65.1 (3)	C71—C73—C74—C76	61.5 (3)
C21—C23—C24—C26	−171.29 (19)	C71—C73—C74—C75	−176.5 (2)
O22—C22—N31—C31	−2.3 (3)	O72—C72—N81—C81	−0.2 (3)
C21—C22—N31—C31	176.57 (19)	C71—C72—N81—C81	−179.37 (18)
C22—N31—C31—C32	−95.4 (2)	C72—N81—C81—C83	117.2 (2)
C22—N31—C31—C33	139.8 (2)	C72—N81—C81—C82	−122.3 (2)
N31—C31—C32—O32	152.7 (2)	N81—C81—C82—O82	−88.0 (2)
C33—C31—C32—O32	−81.4 (3)	C83—C81—C82—O82	33.2 (3)
N31—C31—C32—N41	−30.0 (3)	N81—C81—C82—N91	91.1 (2)
C33—C31—C32—N41	95.9 (2)	C83—C81—C82—N91	−147.7 (2)
N31—C31—C33—C34	−62.2 (3)	N81—C81—C83—C84	−59.5 (3)
C32—C31—C33—C34	171.1 (2)	C82—C81—C83—C84	−178.24 (19)
C31—C33—C34—C36	−55.8 (3)	C81—C83—C84—C85	−58.9 (3)
C31—C33—C34—C35	−179.4 (2)	C81—C83—C84—C86	177.7 (2)
O32—C32—N41—C41	−6.6 (3)	O82—C82—N91—C91	−1.3 (3)
C31—C32—N41—C41	176.13 (19)	C81—C82—N91—C91	179.68 (18)
C32—N41—C41—C42	139.7 (2)	C82—N91—C91—C93	175.07 (19)
C32—N41—C41—C43	−96.0 (2)	C82—N91—C91—C92	54.1 (3)
N41—C41—C42—O42	104.7 (2)	N91—C91—C92—O92	56.2 (3)
C43—C41—C42—O42	−18.8 (3)	C93—C91—C92—O92	−65.7 (3)
N41—C41—C42—N51	−71.0 (2)	N91—C91—C92—N101	−124.2 (2)
C43—C41—C42—N51	165.56 (18)	C93—C91—C92—N101	113.9 (2)
N41—C41—C43—C44	151.11 (19)	N91—C91—C93—C94	166.69 (18)
C42—C41—C43—C44	−87.9 (2)	C92—C91—C93—C94	−72.5 (2)
C41—C43—C44—C49	116.3 (3)	C91—C93—C94—C99	88.8 (3)
C41—C43—C44—C45	−65.2 (3)	C91—C93—C94—C95	−86.1 (3)
C49—C44—C45—C46	0.8 (3)	C99—C94—C95—C96	−0.6 (4)
C43—C44—C45—C46	−177.7 (2)	C93—C94—C95—C96	174.5 (2)
C44—C45—C46—C47	−0.5 (4)	C94—C95—C96—C97	−0.7 (4)
C45—C46—C47—C48	0.0 (4)	C95—C96—C97—C98	1.4 (4)
C46—C47—C48—C49	0.3 (4)	C96—C97—C98—C99	−0.9 (4)
C45—C44—C49—C48	−0.5 (4)	C97—C98—C99—C94	−0.5 (4)
C43—C44—C49—C48	178.0 (2)	C95—C94—C99—C98	1.1 (4)
C47—C48—C49—C44	−0.1 (4)	C93—C94—C99—C98	−173.8 (2)
O42—C42—N51—C51	−0.8 (3)	O92—C92—N101—C101	4.2 (3)
C41—C42—N51—C51	174.80 (17)	C91—C92—N101—C101	−175.37 (18)
O42—C42—N51—C55	173.8 (2)	O92—C92—N101—C105	172.8 (2)
C41—C42—N51—C55	−10.6 (3)	C91—C92—N101—C105	−6.8 (3)
C42—N51—C51—C52	−79.5 (2)	C92—N101—C101—C103	155.1 (2)
C55—N51—C51—C52	105.2 (2)	C105—N101—C101—C103	−15.0 (2)
C42—N51—C51—C53	159.77 (19)	C92—N101—C101—C102	−82.0 (3)
C55—N51—C51—C53	−15.5 (2)	C105—N101—C101—C102	108.0 (2)
N51—C51—C52—O52	176.52 (19)	C11—N11—C102—O102	−1.9 (3)
C53—C51—C52—O52	−68.0 (3)	C11—N11—C102—C101	173.09 (19)
N51—C51—C52—N61	−2.3 (3)	N101—C101—C102—O102	−166.9 (2)
C53—C51—C52—N61	113.2 (2)	C103—C101—C102—O102	−49.6 (3)
N51—C51—C53—C54	32.5 (2)	N101—C101—C102—N11	17.8 (3)
C52—C51—C53—C54	−89.4 (2)	C103—C101—C102—N11	135.1 (2)
C51—C53—C54—O54	77.8 (2)	N101—C101—C103—C104	33.0 (2)
C51—C53—C54—C55	−38.0 (2)	C102—C101—C103—C104	−91.8 (2)
C42—N51—C55—C54	177.27 (19)	C101—C103—C104—O104	77.7 (2)
C51—N51—C55—C54	−7.7 (2)	C101—C103—C104—C105	−39.4 (2)
O54—C54—C55—N51	−90.3 (2)	C92—N101—C105—C104	−178.4 (2)
C53—C54—C55—N51	28.1 (2)	C101—N101—C105—C104	−9.0 (2)
O52—C52—N61—C61	4.2 (3)	O104—C104—C105—N101	−85.6 (2)
C51—C52—N61—C61	−177.09 (18)	C103—C104—C105—N101	29.7 (2)

### cyclo(Val-Leu-Leu-D-Phe-cHyp)2 monohydrate (5) Hydrogen-bond geometry (Å, º)

**Table:**

*D*—H···*A*	H···*A*	*D*···*A*	*D*—H···*A*
N11—H11···O82	2.19	3.012 (2)	159
N31—H31···O62	2.19	2.985 (2)	155
N81—H81···O12	1.98	2.831 (2)	173
N21—H21···O72^i^	2.03	2.870 (2)	164
O54—H54*A*···O42^ii^	1.94	2.759 (2)	177
N71—H71···O102^iii^	2.16	3.000 (2)	166
N91—H91···O22^iii^	2.13	2.949 (2)	159
O104—H10*C*···O32^iv^	2.01	2.823 (3)	170
O1*W*—H1···O54	2.03	2.870 (3)	163
O1*W*—H2···O52^ii^	2.12	2.950 (3)	148

Symmetry codes: (i) −*x*+1, *y*+1/2, −*z*+3/2; (ii) *x*+1/2, −*y*+1/2, −*z*+1; (iii) −*x*+1, *y*−1/2, −*z*+3/2; (iv) −*x*+3/2, −*y*+1, *z*+1/2.
